# Characterisation of HER2‐Driven Morphometric Signature in Breast Cancer and Prediction of Risk of Recurrence

**DOI:** 10.1002/cam4.70852

**Published:** 2025-04-17

**Authors:** N. M. Atallah, S. Makhlouf, M. Nabil, A. Ibrahim, M. S. Toss, N. P. Mongan, E. Rakha

**Affiliations:** ^1^ Translational Medical Science, School of Medicine The University of Nottingham and Nottingham University Hospitals NHS Trust Nottingham UK; ^2^ Department of Pathology, Faculty of Medicine Menoufia University Shebin El‐Kom Egypt; ^3^ Department of Pathology, Faculty of Medicine Assiut University Assuit Egypt; ^4^ Department of Computer Science, Faculty of Medicine Menoufia University Shebin El‐Kom Egypt; ^5^ Department of Pathology Suez Canal University Ismailia Egypt; ^6^ Histopathology Department Royal Hallamshire Hospital, Sheffield Teaching Hospitals NHS Foundation Trust Sheffield UK; ^7^ School of Veterinary Medicine and Sciences University of Nottingham Sutton Bonington UK; ^8^ Department of Pharmacology Weill Cornell Medicine New York New York USA; ^9^ Pathology Department Hamad Medical Corporation Doha Qatar

**Keywords:** artificial neural network, digital image analysis, HER2 oncogenic activity, PAM50 gene assay, response to therapy, risk of recurrence

## Abstract

**Introduction:**

Human epidermal growth factor receptor 2‐positive (HER2‐positive) breast cancer (BC) is a heterogeneous disease. In this study, we hypothesised that the degree of HER2 oncogenic activity, and hence response to anti‐HER2 therapy is translated into a morphological signature that can be of prognostic/predictive value.

**Methods:**

We developed a HER2‐driven signature based on a set of morphometric features identified through digital image analysis and visual assessment in a sizable cohort of BC patients. HER2‐enriched molecular sub‐type (HER2‐E) was used for validation, and pathway enrichment analysis was performed to assess HER2 pathway activity in the signature‐positive cases. The predictive utility of this signature was evaluated in post‐adjuvant HER2‐positive BC patients.

**Results:**

A total of 57 morphometric features were evaluated; of them, 22 features were significantly associated with HER2 positivity. HER2 IHC score 3+/oestrogen receptor‐negative tumours were significantly associated with HER2‐related morphometric features compared to other HER2 classes including HER2 IHC 2+ with gene amplification, and they showed the least intra‐tumour morphological heterogeneity. Tumours displaying HER2‐driven morphometric signature showed the strongest association with PAM50 HER2‐E sub‐type and were enriched with ERBB signalling pathway compared to signature‐negative cases. BC patients with positive HER2 morphometric signature showed prolonged distant metastasis‐free survival post‐adjuvant anti‐HER2 therapy (*p* = 0.007). The clinico‐morphometric prognostic index demonstrated an 87% accuracy in predicting recurrence risk.

**Conclusion:**

Our findings underscore the strong prognostic and predictive correlation between HER2 histo‐morphometric features and response to targeted anti‐HER2 therapy.

## Graphical abstract

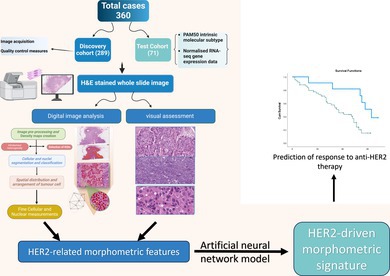

## 1 Introduction

Human epidermal growth factor receptor 2 (HER2) oncogenic activity should ideally correlate with protein over‐expression levels. However, the HER2 positivity definition is based on clinical response to anti‐HER2 therapy and was extended to include breast cancer (BC) with equivocal protein expression (HER2 IHC score 2+) with evidence of *HER2* gene amplification using ISH techniques [1, 2]. Advanced genomic sequencing and pathways studies showed that only 65% of clinically defined HER2‐positive tumours are classified as HER2‐enriched (HER2‐E) using intrinsic molecular sub‐types classification [3, 4, 5], suggesting clinical, pathological and molecular heterogeneity related to HER2 expression level. Therefore, the current definition of HER2 positivity in BC may neither fully reflect the spectrum of oncogenic activity of *HER2* signalling pathways that drive BC growth, differentiation and behaviour nor the clinical and biological heterogeneity within these tumours.

HER2, which is encoded by the *ERBB2* gene, and HER2‐E BC has the highest *ERBB2* mRNA expression, HER2 protein over‐expression, and is associated with higher pathologic complete response (pCR) rates following anti‐HER2‐based regimens [3, 6, 7, 8, 9].

The morphological features of a tumour are the end product of the activity of a specific set of genes working individually or in combination [10, 11, 12, 13, 14]. Correlation between morphometric features and the molecular profiles including molecular aberrations has been previously reported in different tumours including BC [15, 16, 17, 18, 19, 20]. Therefore, accurate assessment of morphometric features of tumour cells could provide insights into molecular and biological cancer signatures and potentially reflect the activity of key driver genes rather than measuring the existence or level of expression of these genes.

Recently, there has been an increasing interest in leveraging the power of artificial intelligence (AI) to identify HER2 status in BC from tumour morphology using the digitalised H&E‐stained slides [21, 22, 23, 24, 25, 26, 27, 28]. Despite being a promising tool, the majority of these AI algorithms utilise an unsupervised approach, without consideration of the biological relevance of the analysed features or HER2 heterogeneity. Current approaches have also failed to explain the molecular basis of their results and have not provided information on how specific predictions are made or elucidated the potential ramifications on therapeutic response [29, 30]. In this work, we hypothesised that further refinement of the clinically diagnosed HER2‐positive BC in an easily validated method which best reflects HER2 oncogenic activity and response to therapy utilising the existing knowledge of the biology and pathology of BC is needed. So, we aimed to assess the role of the digital image analysis tool and visual‐based assessment in defining a set of biologically relevant morphological features that are statistically correlated with HER2 protein over‐expression. Then, we developed a morphometric signature that can reflect HER2‐positive tumours with active oncogenic activity and validated it using HER2‐E PAM50 molecular sub‐type and evaluated the prognostic and predictive validity of the developed signature.

## 2 Materials and Methods

### 2.1 Study Cohorts

#### 2.1.1 Discovery Cohort

**FIGURE 1:**
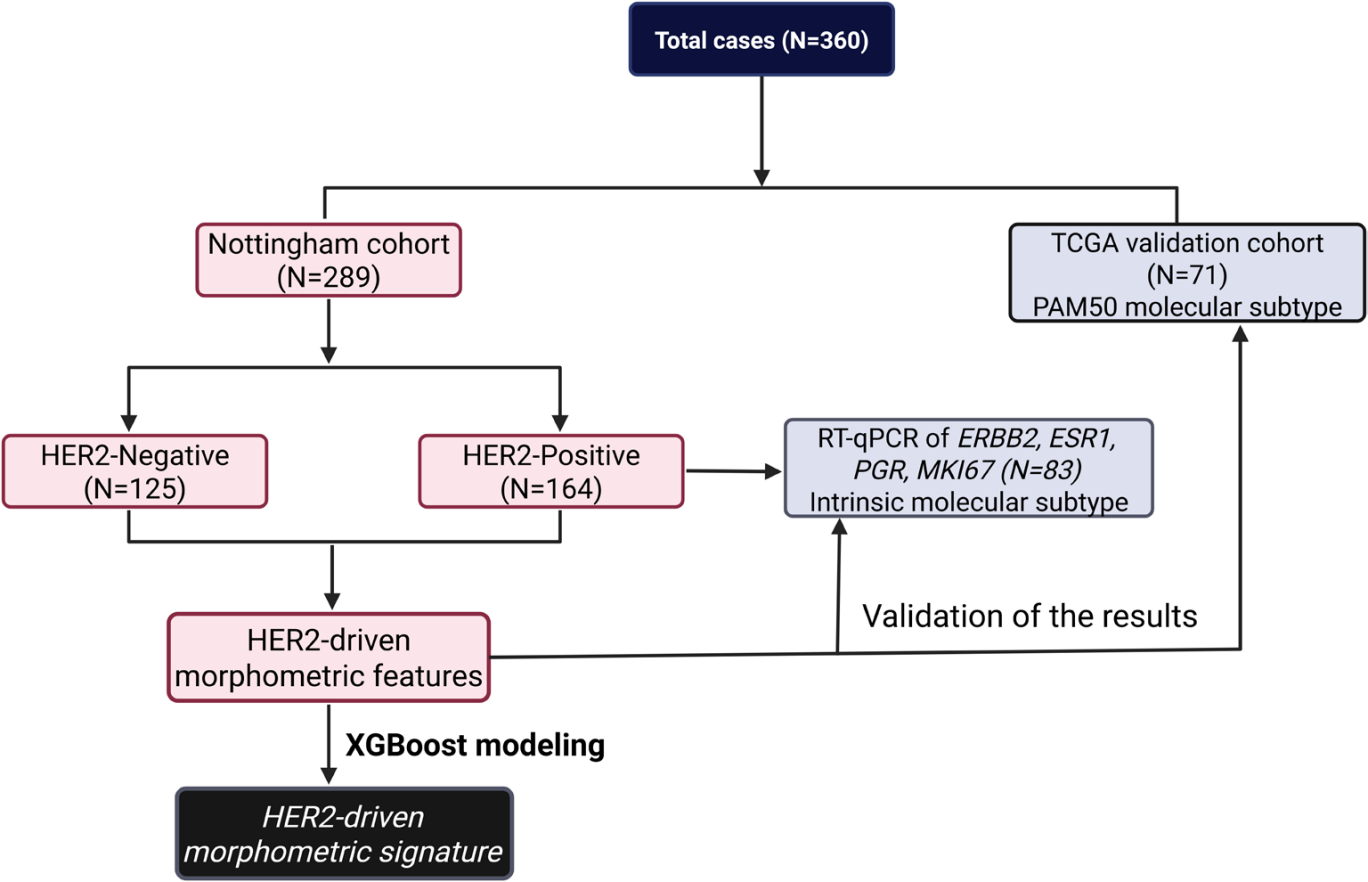
Schematic illustration summarising the characteristics of the study cohort.

A well‐characterised cohort of 289 invasive BC cases presented to Nottingham University Hospitals was investigated. This cohort included two subsets: (1) HER2‐positive cases defined as HER2 IHC score 3+ and IHC score 2+ with *HER2* gene amplification (*n* = 164); (2) a control group of HER2‐negative BC (*n* = 125), including both triple‐negative BC (TNBC) and luminal sub‐types (oestrogen receptor [ER]‐positive/HER2 IHC 0 and 1+) of matched tumour type, grade and stage. All cases in both subsets were invasive breast carcinoma of non‐special type (NST) with tumour grade 2 or 3. Figure 1 represents a schematic illustration of cohort selection and characterisation.

One representative section with adequate tumour content was selected for each case. Clinicopathological data included age at diagnosis, histological tumour grade, tumour size, axillary LN status, lympho‐vascular invasion (LVI), NPI, HR and HER2 status were available from patient records. ER and PR positivity were defined according to ASCO/CAP guidelines, which stipulate a requirement for positive IHC staining in ≥ 1% of the invasive tumour cell nuclei [31]. HER2 staining had been completed on the Ventana Benchmark ULTRA IHC Automated staining system using the Ventana PATHWAY anti‐HER‐2/neu (4B5) rabbit monoclonal ready to use primary antibody in combination with Ventana detection kits as part of patient diagnostics. HER2 scoring was carried out following ASCO/CAP guidelines and UK guidelines [32, 33]. *HER2* gene amplification status, where available, was obtained from patient records.

Treatment regimens and long‐term patient follow‐ups were collected. Patients with HER2‐positive BC were treated with adjuvant anti‐HER2 therapy. Patients' response to therapy was monitored through distant metastasis‐free survival (DMFS).

#### 2.1.2 Test Cohort

Cases from TCGA were included as a test cohort (*n* = 71). Only cases with available virtual histologic slides, HER2 IHC score and PAM50 molecular sub‐type were included. PAM50 molecular sub‐types and their corresponding normalised RNA‐seq gene expression values were downloaded from https://portal.gdc.cancer.gov/. This cohort was utilised as an independent external test cohort to validate the reliability of extracted features and also to assess the enrichment of identified features within HER2 status and HER2‐E molecular sub‐type.

### 2.2 Image Acquisition and Morphometric Digital Image Analysis

**FIGURE 2:**
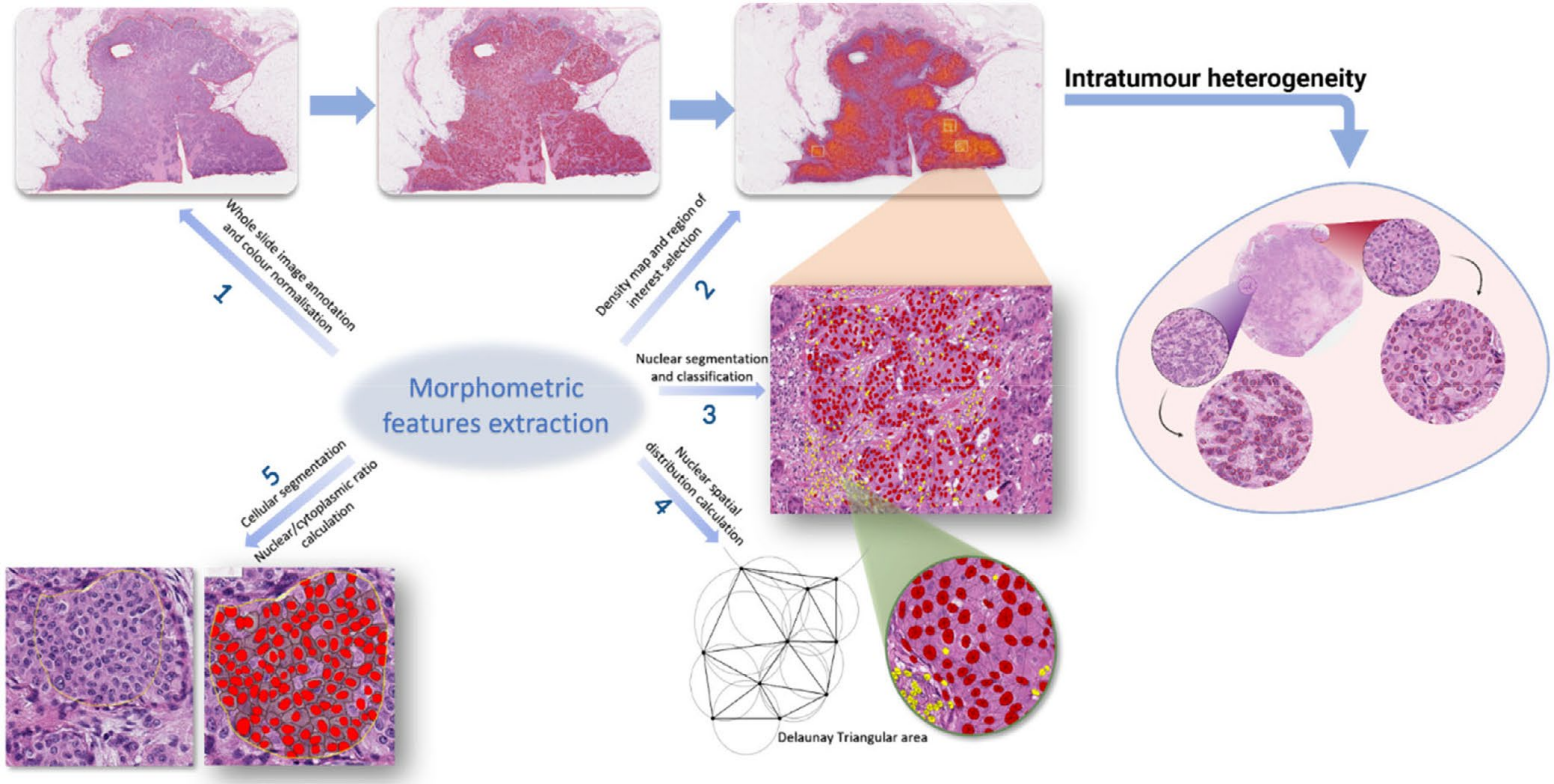
A graph that summarises the process used in image processing to extract morphometric features and evaluate intra‐tumour heterogeneity. Step 1 involved colour normalisation of the entire slide image and tumour cell detection. Step 2 involved creating a density map to identify the regions with the highest tumour density. Density maps were used to select the region of interest (ROI), and three annotation areas of 2000 by 2000 pixels each were made. Step 3: After nuclear annotations and object classifier training, segmented nuclei were divided into nuclei related to tumours and immune cells. Step 4: The Delaunay 2D spatial function was used to calculate the inter‐nuclear distance. Step 5: The nuclear/cell ratio was calculated, and cells were detected using the Cellpose plugin. The variance of morphometric characteristics inside each annotated ROI within the same tumour was used to measure intra‐tumour heterogeneity.

Fresh H&E‐stained sections were scanned at high resolution using the Pannoramic 250 Flash III scanner. All 360 digital images underwent QC and were analysed using QuPath software. The analysis included colour normalisation, stain separation and cellular segmentation to extract biologically relevant morphometric features such as cell count, nuclear dimensions and nuclear/cytoplasmic ratios. Spatial distribution was assessed using the Delaunay 2D model. For objective evaluation of intra‐tumoral heterogeneity in HER2‐positive cases, we computed the variance in the assessed features from annotated areas within each case. Cases were grouped according to ER status. The intra‐tumoral heterogeneity was proven if a significant feature variance was detected between the two groups of HER2‐positive tumours stratified by ER status (Figure 2). Details are mentioned in Appendix S1.

### 2.3 Visual‐Based Assessment

Additional features considering tumour cell architecture and patterns, which could not be analysed through an image analysis approach, were assessed through visual eyeballing assessment. The assessed parameters were based on published literature that addressed different morphological tumour patterns, cellular architecture and arrangement and their clinical significance, if present [34, 35, 36, 37, 38]. To guarantee high reproducibility, the scoring was carried out by three experienced pathologists (N.M.A., S.M. and A.I.), who were totally blind to the clinicopathological and molecular profiles of these cases.

**FIGURE 3:**
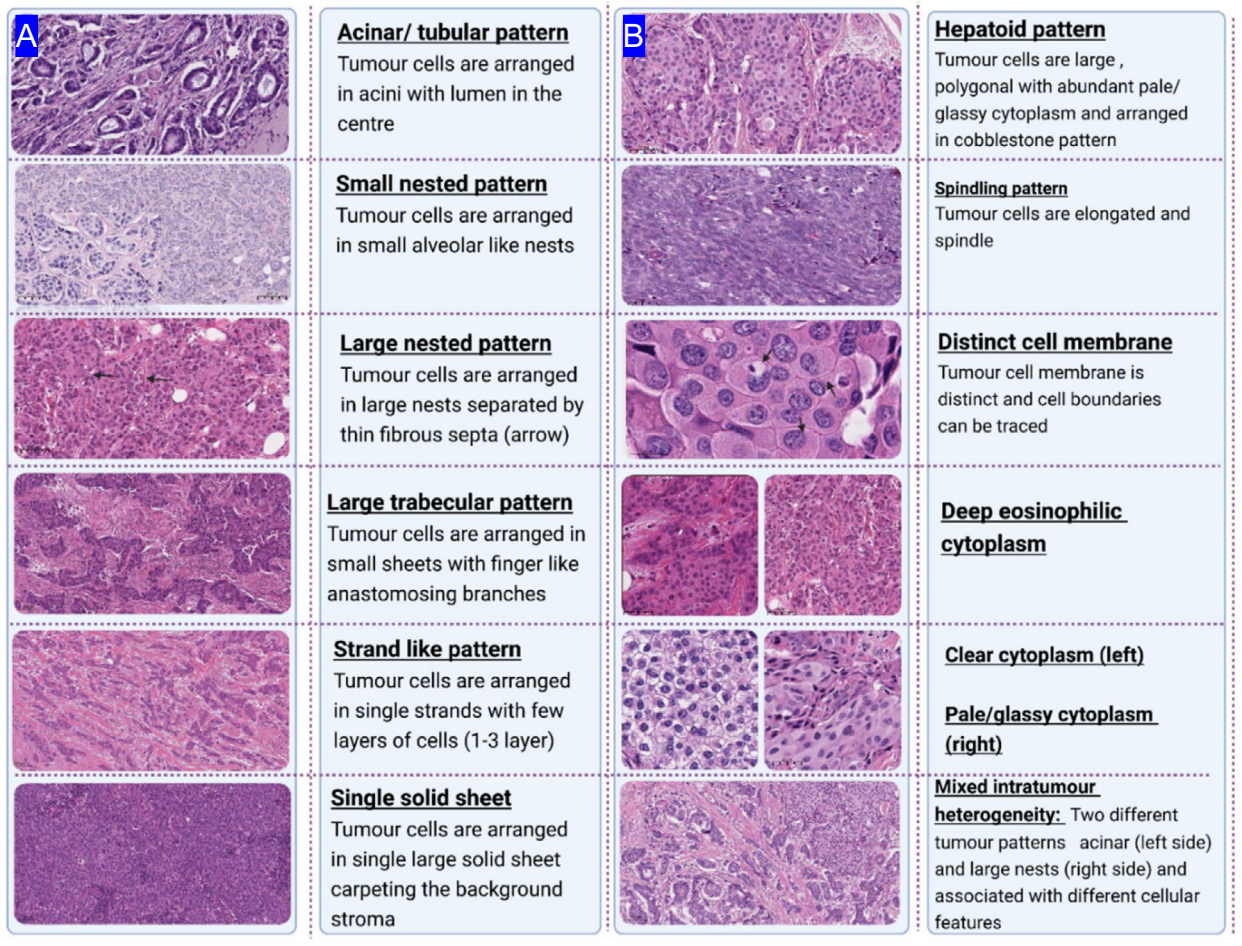
A graph summarising different visually assessed features of tumour cell patterns (A) and architecture (B).

The assessed parameters included:

- Tumour architecture: Six different tumour architectural (cellular arrangement) patterns were evaluated: large solid sheets, tumour strands, small, nested clusters/groups of cells (alveolar‐like pattern), large, nested clusters/groups separated by thin intervening fibrous septa, large trabecular sheets and acinar/tubular pattern. The proportion of each pattern was considered, and the predominant pattern was assigned and included in the analysis (Figure 3A).
- Cellular morphology: Cellular features were categorised based on cell shape, nuclear to cytoplasmic (N/C) ratio, cytoplasmic quality and cell membrane distinction into hepatoid‐like cells (large polygonal and surrounded by abundant pale, glassy cytoplasm and tumour cells are arranged in a cobblestone‐likes structure), spindling with elongated nuclei or no special feature (Figure 3B).
- Cytoplasmic eosinophilia: The intensity and pattern of cytoplasmic staining were assessed as pale/foamy/glassy versus deep eosinophilic and clear cytoplasm.
- Distinction of the cellular membrane (defined or indistinct).
- Intra‐tumour heterogeneity was evaluated by examining the existence of mixed patterns paired with cytoplasmic and membrane characteristics. Mixed architectural heterogeneity refers to tumour architecture heterogeneity accompanied by a shift in cellular pattern. Cases with less than 50% of a single predominant pattern were classified as mixed heterogeneous patterns and given a positive or negative score. Tumours with more than 50% of a single predominant pattern were allocated to this pattern.

### 2.4 Identification of HER2‐Driven Morphometric Features

Out of the total assessed features (both visual and image analysis), we identified the set of features highly associated with HER2 IHC score 3+ tumours, representing the prototype of HER2 protein over‐expression, compared to HER2‐negative and HER2 with equivocal expression tumours. Then we expanded the comparison to include both HER2‐positive sub‐types (3+ and 2+ amplified) and all HER2‐positive cases against HER2−, considering the role of ER status. Data were validated in the HER2‐E molecular sub‐type from TCGA cohort.

### 2.5 Development of a Single HER2‐Driven Morphometric Signature

Then, from the total identified significant set of features, we aimed to develop a single HER2‐driven morphometric signature that could reflect HER2‐positive tumours with evident HER2 oncogenic activity and, hence, a better response to anti‐HER2 therapy. For that, we harnessed the power of ANN through the extreme gradient boosting ‘XGBoost’ model to identify the most effective feature combinations with the highest accuracy in predicting HER2 positivity (Appendix S1).

The significant morphometric features alone and in combination with HR status and HER2 IHC score were used as the input parameters. The obtained signature evaluation metrics included accuracy, precision, recall and F1‐score, and its performance was compared against ERBB2 mRNA using the AUC measuring the sensitivity and the specificity.

The signature with the highest accuracy was then selected for further analysis. Each case had a probability score ranging from 0 to 1; cases were then classified into HER2 morphometric signature positive/negative, according to the cut point predefined using AUC, and its potential clinical significance was assessed through its ability to detect patients who would benefit the most from anti‐HER2 therapy using the DMFS as a surrogate. This model was trained on the discovery cohort, which was split into training (80%) and cross‐validation (20%) sets. The TCGA cohort was used as an independent test cohort, as detailed in Appendix S1.

### 2.6 Differential Gene Expression (DGE) and Pathway Enrichment Analysis

To further test the histo‐morphometric signature score, DGE and pathway enrichment analysis between cases with high and low scores in the TCGA validation cohort were carried out. RNA‐seq data was obtained from TCGA gene expression profile and the DESeq2 tool in version 1.1, IDEP software [39] (http://bioinformatics.sdstate.edu/idep11/) was used for data normalisation and DGE analysis. The significantly differentiated expressed genes were defined as log_2_ fold change (≥ ±1) and FDR < 0.05 between high‐ and low‐score groups. The web‐based gene set enrichment analysis tool (WebGestalt) [40] was used to explore significantly enriched pathways based on the identified DEGs in cases with high HER2‐driven histo‐morphometric signature.

### 2.7 HER2 Risk of Recurrence Prognostic Index

We aimed to establish a link between extracted HER2 morphometric features (covariates) and time to recurrence in HER2‐positive BC patients following adjuvant anti‐HER2 therapy, and accordingly, patients will be divided into high/low risk of recurrence according to the onset of recurrence. We enrolled HER2 morphometric signature both alone and combined with clinicopathological parameters (ER, PR status and tumour size) to develop the optimal prognostic index (clinic‐morphometric) for predicting time to recurrence after anti‐HER2 therapy. Results were compared to models based solely on the HER2 IHC score and on the clinical‐HER2 IHC score, which included the same clinical parameters along with the HER2 IHC score. Patients were categorised into high risk if they developed recurrence early in 5‐year intervals and low risk if no DM occurred in 5 years' time (Appendix S1).

### 2.8 Webtool Application

We developed a web application that offers personalised HER2 signature predictions as a probability score. Utilising the XGBOOST algorithm, the tool analyses features from H&E WSIs to identify morphometric characteristics associated with HER2 expression. This analysis estimates the likelihood of a patient being HER2‐positive, with the probability score ranging from 0 to 1, indicating their potential responsiveness to HER2‐targeted therapies. The following clinical and histologic variables serve as inputs: total detected nuclei, immune cell/tumour cell ratio, nuclear area, nuclear area variance, nuclear length, nuclear maximum diameter, spatial distribution parameters, cell size, N/C ratio and cytoplasm ratio (Figure S1).

### 2.9 Statistical Analysis

Statistical package of social science (IBM‐SPSS) statistical software v. 28.0 (SPSS, Chicago, IL, USA) was used to perform the statistical analyses. We computed the descriptive data (mean, median, range and standard deviations [SD]) for the extracted morphometric features of tumour cells and nuclei. Differences among HER2‐positive and HER2‐negative tumours and other molecular sub‐types were carried out using Mann–Whitney and chi‐square tests. Independent samples *t*‐test was used when comparing means between two independent groups. Inter‐observer agreement was calculated through Fliess kappa test, which determines the level of agreement between two or more raters [41]. Silhouette score was utilised to measure the accuracy of hierarchical clustering analysis. Outcome analysis was assessed using univariate and multivariate Cox regression models and Kaplan–Meier curves and the log‐rank test. For evaluation of the model accuracy, we used the concordance index (*c*‐index). It is a measure of rank correlation between predicted risk scores $\hat{f}$ and observed time points y. For all tests, *p* < 0.05 (two‐tailed) was considered statistically significant.

## 3 Results

### 3.1 Basic Cohort Description

The total number of enrolled cases was 360, including 289 bc cases in the Nottingham discovery cohort and 71 in the TCGA test cohort. Table 1 summarises the baseline patient and tumour characteristics. The mean age of patients was 51 years (range 28–78) and 54.60 years (range 22–80) in the discovery and test sets, respectively.

**TABLE 1:** Baseline patients and tumour characteristics.

Parameters	Discovery set (*n* = 289)		Test cohort (*n* = 71)	
	*N*	%	*N*	%
Age at diagnosis (years)				
< 50	127	43.9	30	42.3
≥ 50	162	56.1	41	57.7
Menopause				
Pre	136	47.1	NA	
Post	153	52.9		
Tumour size (cm)				
< 2.0	152	52.6	21	19.4
≥ 2.0	137	47.4	50	80.6
Tubule formation				
2	34	11.8	19	11.9
3	255	88.2	52	88.1
Pleomorphism				
1	16	5.5	0	0
2	46	15.9	20	15.0
3	227	78.5	51	85.0
Mitosis				
1	124	42.9	8	16.3
2	45	15.6	8	16.3
3	120	41.5	33	67.3
Lympho‐vascular invasion				
No	183	63.3	42	59.2
Yes	106	36.7	29	40.8
Lymph node status				
Negative	155	53.6	NA	
Positive	134	46.4		
HER2 IHC status				
Negative	125	43.3	27	38
Positive	164	56.7	44	62
HER2 IHC scores				
0/1+	95	32.8	15	21
2+/non‐ISH amplified	30	10.1	10	14
2+/ISH amplified	46	16.3	19	27
3+	118	40.8	27	38
ER status				
Negative	136	47.1	35	48.5
Positive	153	52.9	36	51.5
PR status				
Negative	179	61.9	41	58.3
Positive	110	38.1	30	42.7
PAM50 molecular sub‐type				
Luminal A	NA		5	7.0
Luminal B			20	28.2
HER2 enriched			27	38.0
Basal like			18	25.4
Normal like			1	1.4
NPI risk group				
Low risk	24	8.3	NA	
Intermediate risk	193	66.8		
High risk	72	24.9		
Adjuvant anti‐HER2 therapy				
No	26	18	0	0.0
Yes	118	82	36	100

*Note:* Some cases are missing. Grey shades is just a format to differentiate between both study cohorts.

Abbreviations: ER, oestrogen receptor; IHC, immunohistochemical expression; NPI, Nottingham prognostic index; PR, progesterone receptor.

### 3.2 Characterisation of HER2‐Driven Morphometric Features

#### 3.2.1 Image Analysis

In total, ~914,024 nuclei were assessed, including both tumour and immune cells. The total number of the extracted high throughput subcellular morphometric features was 40; however, only 18 features have meaningful biological and statistical relevance, demonstrated significant associations with HER2‐positive compared to HER2‐negative tumours, and were employed for all subsequent analyses (Table S1, Tables 2 and 3). These features included tumours with lower tumour cell density, larger nuclear and cellular area, open‐phase nuclear chromatin, high variability in nuclear size and tumours with widely spaced, less overlapped tumour nuclei (*p* < 0.001).

**TABLE 2:** Correlation between different HER2 immunohistochemical scores and cyto‐morphometric features in the discovery cohort.

	HER2 IHC 3+		*p* ^a^	HER2 IHC 2+		*p* ^b^	*p* ^c^	HER2‐negative		*p* ^d^
	ER‐negative (*n* = 60)	ER‐positive (*n* = 58)		ISH‐positive (*n* = 46)	ISH‐negative (*n* = 30)			ER‐positive/HER2 0/1+ (*n* = 46)	Triple‐negative (*n* = 49)	
	Mean ± SD	Mean ± SD		Mean ± SD	Mean ± SD			Mean ± SD	Mean ± SD	
Average total detected nuclei	2802 ± 689.2	3192 ± 904.9	**0.009**	3278 ± 998	3203.1 ± 1560	0.13	0.32	3830.3 ± 1116	4265.5 ± 685	**< 0.001**
Average tumour nuclei	1861 ± 550.1	2218 ± 577.9	**0.001**	2191.1 ± 656	2236.4 ± 1083	0.20	0.65	2720.9 ± 931.1	2564.5 ± 186	**< 0.001**
Average immune cells	934 ± 537	934 ± 735.6	0.8	1063.3 ± 868	966 ± 712	0.99	0.66	1061.2 ± 879.2	1583.1 ± 192	**0.045**
Immune cells/total cells ratio	32.9 ± 13.0	28.1 ± 13.4	0.08	30.6 ± 15.1	28.1 ± 12.8	0.91	0.60	27.3 ± 17.1	35.4 ± 17.1	0.828
Nuclear area (μm^2^)	64.5 ± 12.9	57.9 ± 9.6	**0.004**	60.5 ± 16.3	58.3 ± 10.6	0.33	0.85	50.9 ± 7.7	58.4 ± 10.0	**< 0.001**
Nuclear area standard deviation	24.5 ± 10.6	19.4 ± 6.9	**0.002**	21.4 ± 10.6	19.8 ± 7.2	0.54	0.50	17.1 ± 5.5	21.4 ± 6.8	**0.009**
Nuclear area variance	713.5	420.6	**0.005**	572.4	446.2	0.54	0.50	321.2	491.2	**0.009**
Nuclear length (μm)	29.1 ± 2.7	27.8 ± 2.2	**0.006**	28.2 ± 3.1	27.8 ± 2.5	0.41	0.77	26.4 ± 3.1	27.5 ± 2.2	**< 0.001**
Nuclear max diameter (μm)	10.7 ± 0.05	10.1 ± 0.8	**0.006**	10.4 ± 1.2	10.3 ± 0.94	0.52	0.67	9.8 ± 2.2	10.0 ± 0.9	**< 0.001**
Nuclear min diameter (μm)	7.5 ± 0.6	7.23 ± 0.61	**0.013**	7.2 ± 0.8	7.2 ± 0.61	0.26	0.87	6.8 ± 0.5	7.1 ± 0.5	**< 0.001**
Nuclear haematoxylin optical density	0.35 ± 0.1	0.39 ± 0.1	0.74	0.37 ± 0.1	0.34 ± 0.1	0.72	0.19	0.68 ± 0.8	0.4 ± 0.1	**< 0.001**
Delaunay: mean distance	19.4 ± 32.	17.9 ± 2.4	**< 0.001**	18.4 ± 3.5	18.7 ± 2.7	0.18	0.43	15.7 ± 2.4	16.5 ± 2.3	**< 0.001**
Delaunay: mean triangle area	145.3 ± 33.4	122.8 ± 33.7	**< 0.001**	133.2 ± 51.2	135.6 ± 37.6	0.22	0.46	96.7 ± 24.8	107.7 ± 32.8	**< 0.001**
Delaunay: max triangle area	228.6 ± 57.3	197.47 ± 64.2	**0.003**	213.9 ± 96.8	223.8 ± 73.1	0.24	0.29	156.9 ± 43.6	163.7 ± 63.6	**< 0.001**
Cell area (μm^2^)	129.0 ± 35.0	121.1 ± 34.0	0.22	117.1 ± 45.9	117.9 ± 29.6	**0.01**	0.33	94.1 ± 20.5	NA	**< 0.001**
Nuclear/Cell ratio	0.51 ± 0.09	0.50 ± 0.1	0.51	0.54 ± 0.09	0.51 ± 0.11	**0.01**	0.13	0.55 ± 0.08	NA	**0.095**
Cytoplasm area	65.5 ± 31.9	63.8 ± 33.4	0.77	56.6 ± 35.9	60.4 ± 27.9	**0.007**	0.19	43.1 ± 17.1	NA	**0.001**
Nuclear/Cytoplasmic ratio	1.1 ± 0.49	1.08 ± 0.6	0.80	1.28 ± 0.45	1.12 ± 0.48	**0.01**	0.12	1.3 ± 0.4	NA	**0.072**

*Note:* Independent samples *t*‐test was used when comparing means between two independent groups. Bold values are for significant *p* values.

Abbreviations: ER, oestrogen receptor; ISH, in situ hybridisation.

^a^ *p* value between HER2 IHC 3+/ER‐negative and HER2 IHC 3+/ER‐positive.

^b^ *p* value between HER2 IHC 3+ and HER2 IHC 2+/ISH+.

^c^ *p* value between HER2 IHC 2+/ISH+ and HER2 IHC 2+/ISH−.

^d^ *p* value between HER2‐positive (HRE2 IHC 3+ and 2+/ISH positive) and HER2‐negative.

**TABLE 3:** Description of the biologically relevant morphometric features in TCGA cohort.

Morphometric features	HER2‐E	Luminal B	Luminal A	TNBC^a^
	Mean ± SD/Median (range)	Mean ± SD/Median (range)	Mean ± SD/Median (range)	Mean ± SD/Median (range)
Average total detected nuclei/case	3217 (1711–11,097)	3593 (1847–12,401)	3325 (2086–3989)	3495 (1732–4840)
Average tumour nuclei/case	1935 (1144–7240)	2446 (871–9688)	1491 (1034–2019)	2383 (1233–3817)
Average immune cells/case	1292 (226–3857)	1146.9 (307–2713)	1834 (1052–2391)	1112 (361–2354)
Immune cells/total cells ratio	0.38 ± 0.17	0.33 ± 0.15	0.54 ± 0.05	0.32 ± 0.15
Nuclear area (μm^2^)	62.5 ± 11.9	61.5 ± 9.4	47.5 ± 2.1	56.37 ± 8.6
Nuclear area SD	24.8 ± 9.9	21.8 ± 6.1	15.8 ± 4.6	20.03 ± 6.6
Nuclear area variance	748.2	513.8	268.4	441.5
Nuclear length (μm)	28.9 ± 2.6	28.7 ± 2.0	25.5 ± 0.71	27.6 ± 2.2
Nuclear max diameter (μm)	10.8 ± 1.02	10.6 ± 0.72	9.4 ± 0.35	10.2 ± 0.9
Nuclear min diameter (μm)	7.3 ± 0.67	7.3 ± 0.63	6.46 ± 0.11	7.02 ± 0.15
Nuclear haematoxylin OD	0.48 ± 0.16	0.52 ± 0.18	0.47 ± 0.15	0.42 ± 0.15
Delaunay: mean distance	21.4 ± 3.8	17.6 ± 1.9	17.5 ± 2.5	17.14 ± 1.8
Delaunay: mean triangle area	147 ± 51	128.1 ± 25.0	127.6 ± 39.0 1	113.3 ± 24.5
Delaunay: max triangle area	218 ± 68.6	198.6 ± 47.1	176.2 ± 57.1	169.02 ± 39.2
Cell area (μm^2^)	139 ± 36.07	118.3 ± 32	77.9 ± 13.8	99.5
Nuclear/Cell ratio	0.47 ± 0.12	0.52 ± 0.18	0.6230.4 ± 0.13	0.3742
Cytoplasm area	77.3 ± 34.3	57.0 ± 31.8	30.4 ± 13.3	46.4280
Nuclear/Cytoplasmic ratio	1.01 ± 0.66	1.5 ± 1.1	1.8 ± 0.8	1.4206

Abbreviations: HER2‐E, HER2‐enriched; OD, optic density; SD, standard deviation; TNBC, triple‐negative breast cancer.

^a^ TNBC includes both basal and normal‐like sub‐types.

**FIGURE 4:**
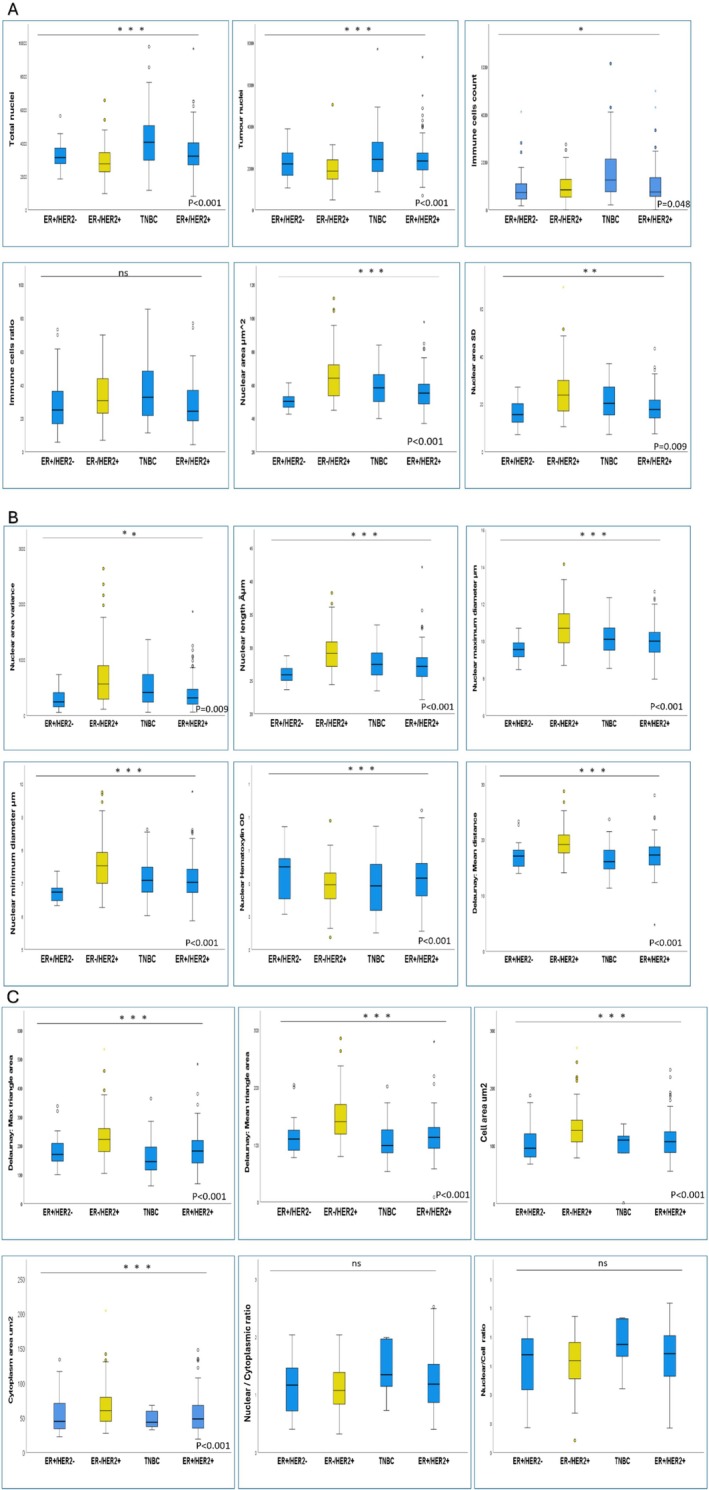
Box plot chart summarising the correlation between the significant morphometric features in the discovery cohort and different HER2 classes.

In both discovery and test cohorts, categorisation of HER2‐positive cases according to HER2 IHC scores, tumours with HER2 IHC3+ showed the highest enrichment of the identified morphometric features and were significantly different from HER2‐positive tumours with borderline protein expression. Within HER2 2+ tumours, no statistically significant difference in the assessed morphometric features was identified between *HER*2 gene amplified and non‐amplified (Table 2). HER2‐positive/ER‐negative tumours were more enriched with HER2‐driven morphometric features compared to HER2‐positive/ER‐positive tumours (Table 2 and Figure 4). 

**FIGURE 5:**
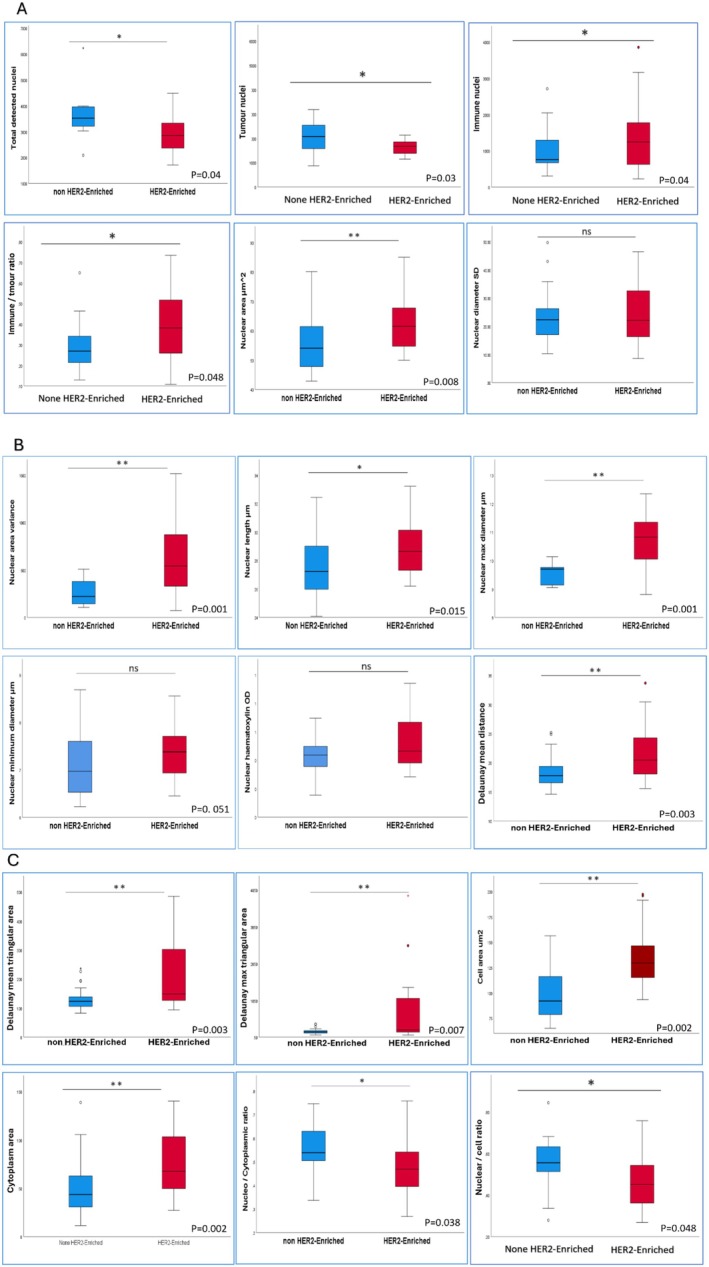
Box plot showing the correlation between the pre‐identified morphometric features and PAM50 HER2‐enriched molecular sub‐type in the test cohort.

In the TCGA cohort, HER2‐E sub‐type was significantly associated with most of the HER2‐driven morphometric features (15/18) compared to clinically HER2‐positive/non‐HER2‐E on the PAM50 assay (Figure 5).

**FIGURE 6:**
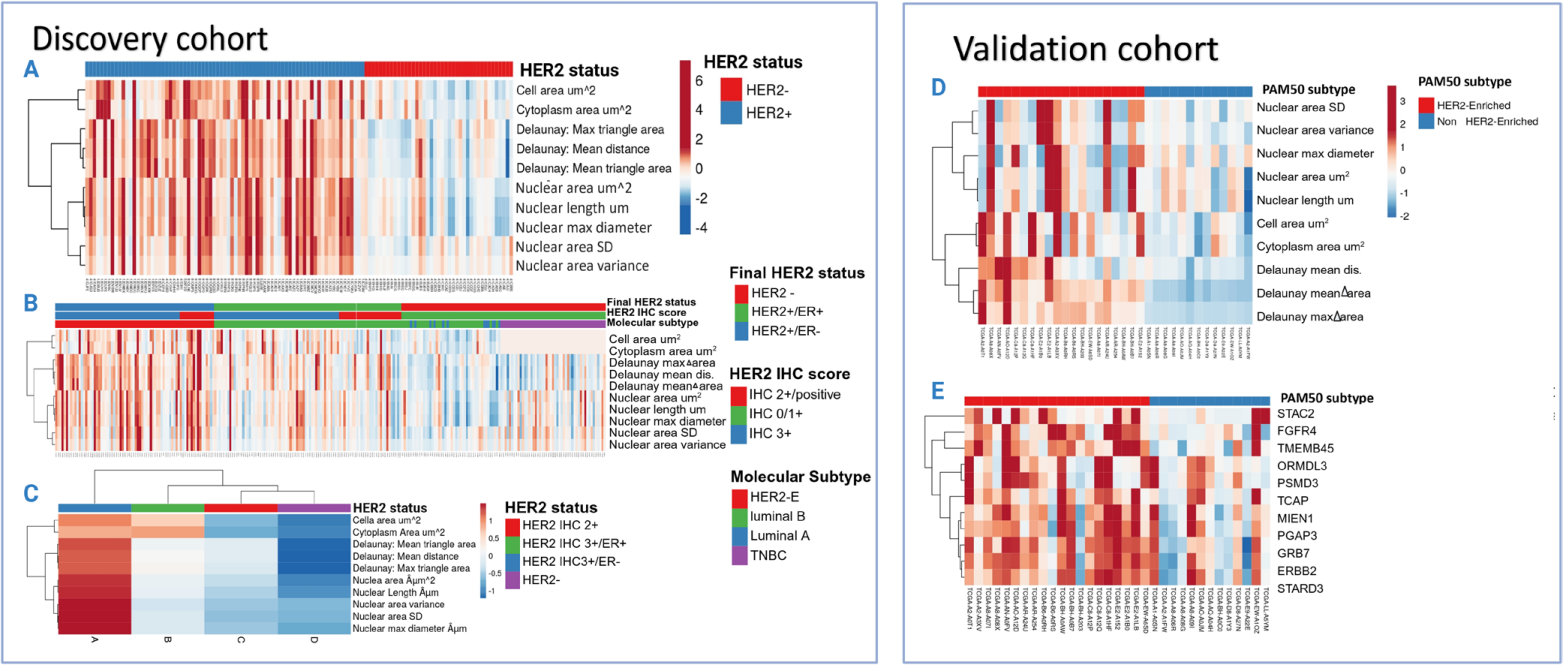
A heat map showing how cases in the discovery and test cohorts were grouped hierarchically based on HER2‐driven morphometric characteristics. (A) The morphometric features allowed for a distinct separation of HER2 positive and negative. (B) The panel shows the same cohort split up by ER status and HER2 IHC score. When compared to other classes, HER2 IHC3+/ER‐negative tumours had a much higher enrichment of morphometric characteristics. (C) Shows how the instances are grouped according to the morphometric feature's summed means for each class. Similar to HER2 oncogenic signalling genes (E), HER2‐driven morphometric characteristics in the test cohort effectively segregated PAM50 molecular sub‐type into HER2‐E and non‐HER2‐E (D).

The hierarchical clustering of the cases in the discovery cohort using HER2‐driven morphometric features as the clustering input yielded clearly defined clusters with the highest enrichment of the features obtained in HER2 IHC3+/ER‐negative, and the silhouette score reached 0.65. Similar results were observed in the TCGA cohort, where the highest enrichment was seen in the HER2‐E PAM50 molecular sub‐type with a 0.53 clustering score and compared favourably to the 0.40 clustering score obtained when applying HER2 oncogenic signalling pathway genes [42] as the clustering input instead of the morphometric features (Figure 6). For better viewing of the graph, we included features that are positively correlated with HER2‐positive tumours.

**FIGURE 7:**
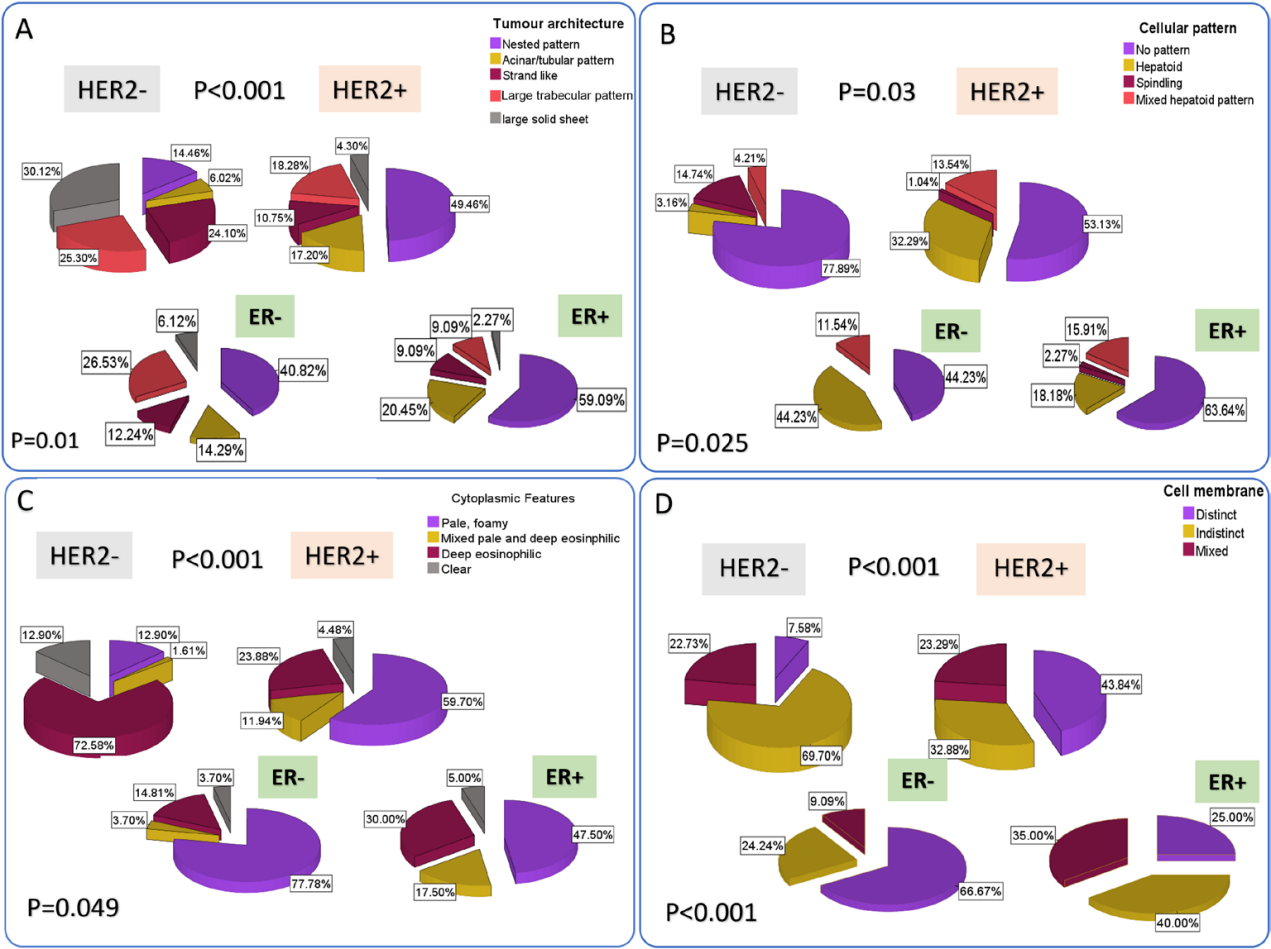
Pie chart summarising the distribution of the visually assessed features between HER2‐positive and HER2‐negative tumours. HER2‐positive tumours are significantly associated with nested tumour architecture (A), hepatoid‐like cellular pattern (B) with pale eosinophilic cytoplasm (C) and distinct cell membrane (D). Within HER2‐positive tumours, a significant difference was also seen in the visually assessed features between ER− and ER+ cases. ER−, ER‐negative; ER+, ER‐positive; HER2−, HER2‐negative; HER2+, HER2‐positive.

#### 3.2.2 Correlation Between Morphometric Features and Clinic‐Pathological and Molecular Parameters

In HER2‐positive tumours, larger inter‐nuclear spatial distribution parameters, larger cell and cytoplasmic areas were significantly associated with an increased number of metastatic LNs. Poor NPI was significantly associated with high tumour cell density and a less immune/tumour cell ratio. Higher ER% in HER2‐positive cases was significantly associated with decreased nuclear‐related dimensions (area, length, diameter and variance) (*r* = −0.4, *p* = 0.001) and decreased inter‐cellular spatial distances (*r* = −0.34, *p* = 0.002) (Figure S2A).

In the test cohort, there was a significant positive association between *ERBB2* mRNA and larger inter‐nuclear spatial distance (*r* = 0.55, *p* < 0.001), features related to larger cell and cytoplasmic size (*r* = 0.43, *p* = 0.01 and *r* = 0.32, *p* = 0.02), respectively, and less nuclear/cytoplasmic ratio (*r* = −0.29, *p* = 0.03) as presented in the correlation matrix (Figure S2B). In contrast, high *ESR1* mRNA level was significantly associated with decreased nuclear‐related dimensions (*p* = 0.01) and decreased inter‐cellular spatial distances (*p* = 0.02).

### 3.3 Visual Assessment of Tumour Features

Seventeen visually assessed features were evaluated, encompassing detailed architectural and cellular morphologies that were not analysed using image analysis. The overall inter‐observer agreement was 0.75 (excellent), which entailed 0.79 for tumour architecture, 0.82 for cellular patterns and 0.71 for both cytoplasmic eosinophilia and cell membrane distinction.

Four main features were significantly associated with HER2 positivity. Regarding tumour architecture, the nested tumour pattern (both small and large) (50%) was the main one that significantly associated with HER2‐positive tumours (*p* < 0.001), compared to the large trabecular tumour pattern (18%) and large solid sheets of tumour cells. The latter was more predominant in TNBC (4% vs. 30% for HER2‐positive and TNBC, respectively). For cellular patterns, a significant association was found between HER2‐positive tumours and cells exhibiting a polyhedral shape (*p* = 0.03), abundant pale and foamy cytoplasm (*p* < 0.001) and cells with distinct cellular membranes (*p* < 0.001), compared to HER2‐negative tumours (Figure 7).

Through visual‐based assessment of intra‐tumour heterogeneity, 35% of HER2‐positive tumours have mixed tumour architectures associated with heterogeneous cellular patterns ‘mixed architecture heterogeneity’, 12% showed mixed cytoplasmic features, 23% showed mixed cell membrane pattern. Upon stratifying HER2‐positive cases according to ER status, intra‐tumour mixed architecture heterogeneity and mixed cell membrane pattern, were significantly associated with HER2‐positive/ER‐positive compared to HER2‐positive/ER‐negative tumours (*p* = 0.007, *p* < 0.001 and *p* = 0.007, respectively).

Assessment of the features variance among different annotated tumour areas within the same case revealed that the features variance within HER2‐positive/ER‐negative tumours was significantly less than that corresponding within HER2‐positive/ER‐positive regarding nuclear area median difference (2.1 vs. 7.5, *p* = 0.02), nuclear area SD median difference (1.02 vs. 4.3, *p* = 0.003) and nuclear area variance mean difference (68.3 vs. 122, *p* = 0.017) (Figure S3).

### 3.4 HER2‐Driven Morphometric Signature and Response to Anti‐HER2 Therapy

**FIGURE 8:**
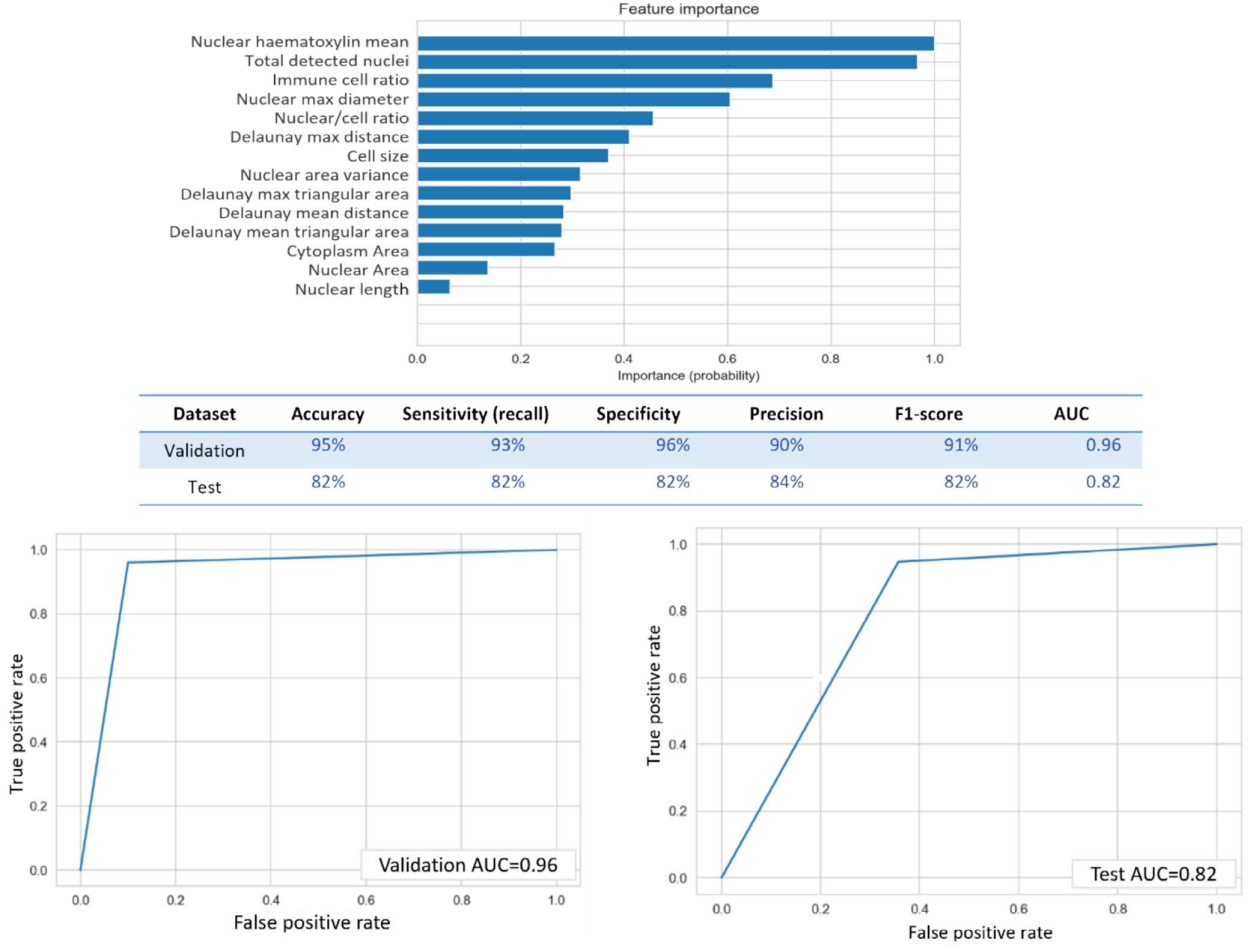
HER2 morphometric signature component and its performance. The receiver operating characteristic curves (ROC) highlight the signature accuracy in the cross‐validation and external test cohort.

We ended up with 22 (18 image analysis based and 4 visually assessed) features that are significantly correlated with HER2‐positive tumours. For the development of a morphometric signature that can be used in clinical practice, a gradient boosting classifier was employed. The model was trained using continuous, non‐parametric image analysis features only, as incorporating visually assessed categorical features could potentially affect the model's performance. This signature was able to predict HER2‐positive tumours with 95% accuracy (AUC = 0.96) in the cross‐validation set and 83% (AUC = 0.82) in the external test cohort (Figure 8), in comparison to *ERBB2* mRNA (AUC = 0.80). Out of the 18 cyto‐morphometric features, the model selected 14 based on their relevance and contribution to improving model accuracy, as demonstrated in Figure 8. The HER2 morphometric signature is characterised by larger nuclear and cellular areas, increased nuclear size variability and wider inter‐nuclear spacing, reflecting an aggressive tumour phenotype associated with high HER2 activity.

**FIGURE 9:**
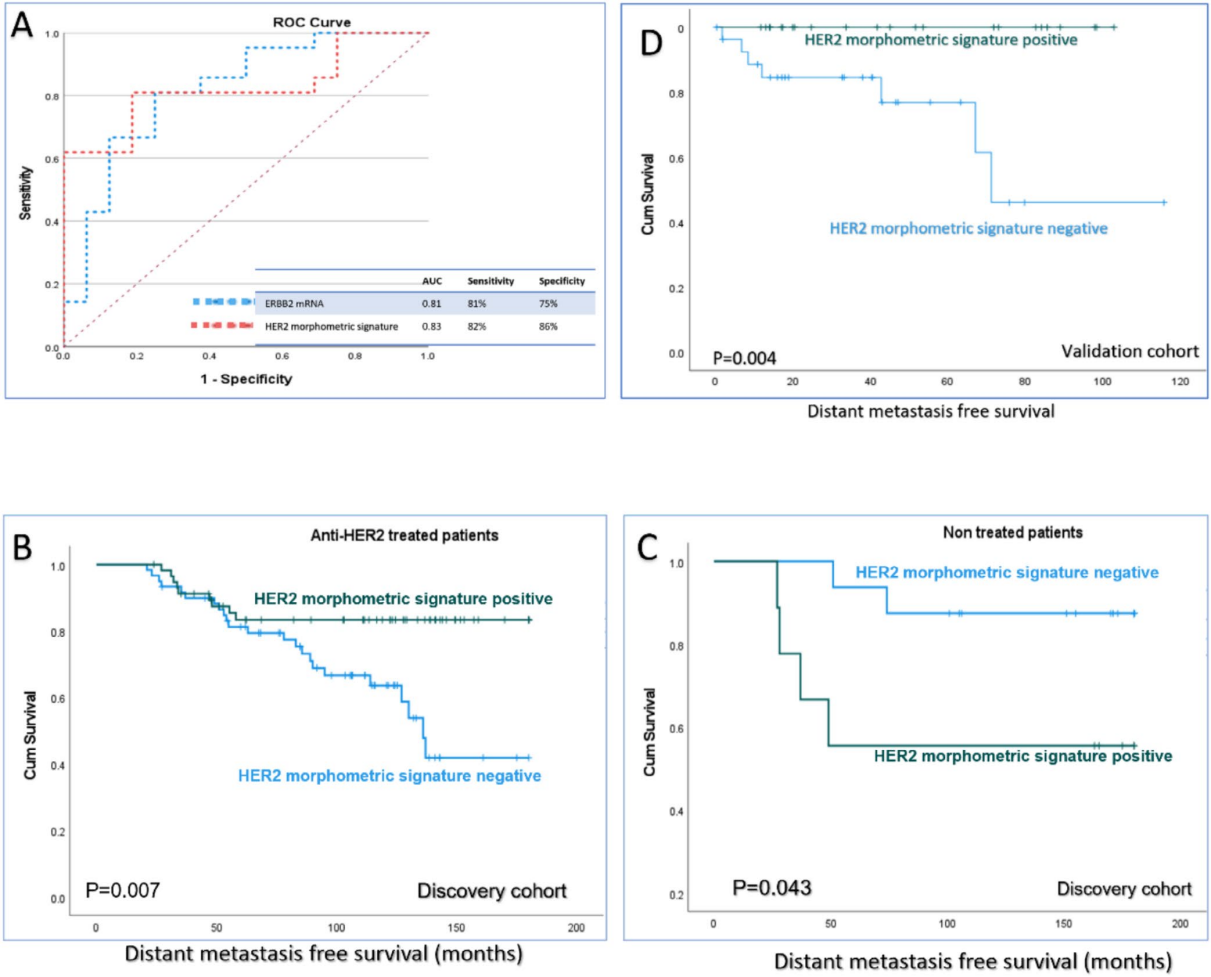
A graph demonstrating the performance and clinical significance of the HER2 morphometric signature. (A) Receiver operating characteristics (ROC) curve comparing the HER2 morphometric signature and *ERBB2* mRNA in the prediction of the HER2‐E sub‐type. (B, D) Kaplan–Meier curve showing the impact of HER2 morphometric signature‐positive patients on the response to anti‐HER2 therapy with prolonged distant metastasis‐free survival in discovery and test cohorts, respectively. While in non‐treated HER2‐positive patients (C), HER2 morphometric signature‐positive tumours had a higher frequency of distant metastasis.

According to the probability score obtained from the model, cases were divided into either morphometric signature positive or negative. HER2‐positive patients treated with adjuvant anti‐HER2 therapy who had BC with positive HER2‐morphometric signature had significantly longer DMFS (*p* = 0.007, *p* = 0.004 in the discovery and test set, respectively) (Figure 9). Contrasting this, the subset of HER2‐positive patients who did not receive anti‐HER2 therapy showed a significantly higher risk of recurrence when their BC displayed a positive HER2 morphometric signature (*p* = 0.048).

In a multivariate analysis, HER2 morphometric signature was an independent predictor of prolonged DMFS post‐adjuvant anti‐HER2 therapy when adjusted for tumour size, LN status and LVI and HER2 IHC score (HR: 0.4, *p* = 0.026, 95% CI: 0.18–0.89).

Pathway enrichment analysis revealed enrichment with the ERBB signalling pathway (FDR = 0.03) with upregulation of the ERBB2 gene in the positive HER2‐driven morphometric signature class (Table 4).

**TABLE 4:** Pathway enrichment analysis of HER2‐morphometric signature‐positive versus HER2‐morphometric signature‐negative cases.

Gene set	Description	Size	FDR
hsa01522	Endocrine resistance	98	0.0028299
hsa05224	Breast cancer	147	0.0048011
hsa05205	Proteoglycans in cancer	201	0.0081827
hsa05213	Endometrial cancer	58	0.027888
hsa05223	Non‐small cell lung cancer	66	0.027888
hsa04917	Prolactin signalling pathway	70	0.027888
hsa01521	EGFR tyrosine kinase inhibitor resistance	79	0.030446
hsa04012	ErbB signalling pathway	85	0.030836
hsa05215	Prostate cancer	97	0.035670
hsa05200	Pathways in cancer	526	0.042924

### 3.5 HER2‐Positive BC Patient Risk of Recurrence Prognostic Index

HER2 IHC score (3+ vs. 2+ amplified) based prognostic index shows moderate accuracy (AUC = 0.47) in predicting the risk of patients' recurrence, while the morphometric features achieved an AUC of 0.75. When the HER2‐driven morphometric features were combined with ER and PR status (clinico‐morphometric prognostic index) this achieved the highest accuracy in prediction of the risk of early recurrence in HER2‐positive BC patients with *c*‐index = 0.81, AUC = 0.87 in the test cohort. This was better than the combined HER2 IHC score plus ER and PR status score, which achieved an AUC of 0.61 (Figure S4).

## 4 Discussion

HER2‐positive BC constitutes approximately 15% of all BC cases [43]. HER2‐E BC is characterised by the highest levels of *ERBB2* mRNA and total HER2 protein, suggesting that this group has the highest activation of the HER2 signalling pathway [4, 44, 45]. However, not all clinically HER2‐positive cases are HER2‐E sub‐type, and only 65% fall in that category [4, 44]. Currently, every HER2‐positive BC patient is eligible for anti‐HER2 therapy regardless of HER2‐E status; yet, the response rate varies, with 20% of patients experiencing recurrence and metastasis post‐adjuvant therapy [29, 30]. Identification of HER2‐positive tumours with active HER2 oncogenic activity through a clinically applicable and cost‐effective tool is mandatory for prediction of patients who would benefit the most from HER2 signalling pathway targeting therapy, both in the neoadjuvant and adjuvant settings [45, 46, 47, 48, 49]. Building upon the established morpho‐molecular signature across various tumours [15, 16, 17, 18, 19, 20], our aim was to delineate a collection of clinically pertinent histo‐morphometric features. These features aim to reflect the extent of HER2 signalling pathway activity and establish connections between these features and response to anti‐HER2 targeted therapy, incorporating digital image analysis and culminating in the development of a clinically feasible and cost‐effective tool. To the best of our knowledge, there have been no prior studies aimed at characterising the activity of the HER2 oncogene using the tumour morphological features and response to therapy.

We were able to identify a set of features highly associated with HER2‐positive tumours as well as HER2‐E intrinsic sub‐type. Those features included large polygonal cells with larger nuclei, open‐phase nuclear chromatin that was evident through the pale nuclear haematoxylin optical density, more abundant pale eosinophilic cytoplasm which reduced the N/C ratio, less tumour cell density, a higher immune/tumour cell ratio and wider inter‐nuclear spatial distances. This allies with previous reports that stated that the active *HER2* signalling pathway stimulates the downstream cascade increasing cell proliferation and modulation in chromatin structure to yield an open‐phase chromatin [50].

We also found that HER2‐positive tumour cells tend to have distinct cellular membranes and are arranged in a small nested/alveolar‐like pattern rather than single solid sheets that were predominant in TNBC. This was previously reported by Denisov et al. who performed pathway analysis of transcripts that were differentially expressed in the different morphological structures of breast tumours and found that tumours arranged in small nests showed a more considerable association with the *ERBB2* pathway [37]. TNBC cells were reported to have a syncytial pattern with ill‐defined cell borders compared to HER2‐positive and luminal tumours [51], which was also evident in our study.

HER2‐driven morphometric features were highly predominant in HER2 IHC 3+/ER‐negative BC cases compared to HER2 IHC 3+/ER‐positive and HER2 IHC 2+ with evidence of *HER2* gene amplifications. This agrees with previous reports that stated HER2 protein over‐expression (IHC score 3+) is the key driver of *HER2* oncogenic activity and is highly enriched with *HER2* signalling pathway genes compared to borderline HER2‐positive BC with evidence of gene amplification [42, 52].

The distribution of HER2‐E sub‐type and activity of *HER2* oncogenic signalling seem to be heavily influenced by ER status, with HER2‐E sub‐type representing only 30% of molecular sub‐types within HER2‐positive/HR‐positive BC [44, 46, 48, 53, 54], some authors even claimed that HER2‐E sub‐type is predominantly ER‐negative [53]. According to St. Gallen Expert Consensus, HER2‐positive tumours are divided into two classes: a Luminal B‐like sub‐type that features ER and/or PR expression and a HER2‐E‐like sub‐type, which does not express both HR [55]. Several clinical trials and studies highlighted the bidirectional cross‐talk between HER2 and ER when both receptors are expressed in BC cells [54, 56, 57, 58, 59, 60, 61, 62], and these reports concluded that HER2‐positive tumours differ in main clinicopathological features and natural history of disease depending on HR expression. More specifically, HER2‐positive/ER‐negative tumours are more likely to present with high histologic grade and higher tumour stage, less likely to first relapse in bone, and more likely to recur in brain than HER2‐positive/HR‐positive tumours [63].

HER2‐positive/ER‐positive BC is difficult to treat. There is not one treatment or set of treatments that works well for every patient with this sub‐type. Although the cancer may change course during treatment due to pathway interaction and cross‐talk, it can be very helpful to determine the biological driver of the specific tumour (i.e., whether ER or HER2 signalling is dominating and driving the growth and advancement of the tumour). It has been demonstrated that cells with acquired resistance to anti‐HER2 therapy exhibit elevated expression of ER and its downstream targets [64, 65].

Furthermore, HER2 over‐expression affects endocrine therapy responsiveness both to tamoxifen and to oestrogen deprivation by aromatase inhibitors and ovarian suppression in pre‐menopausal women [64, 66, 67, 68, 69, 70, 71, 72].

Combining hormone therapy with an anti‐HER2 agent has proven beneficial to some specific HER2‐positive patients [73, 74], particularly those who have high ER expression. Some ER‐positive/HER2‐positive tumours behave more like the luminal A sub‐type (i.e., ER‐driven cancer) and others as HER2‐E tumours (HER2‐driven cancer) or a combination of both, which requires a multi‐pronged targeted blockade of both ER and HER2 pathways [42].

The identified morphometric signature reflected both HER2‐E, representing the oncogenic activity of the *HER2* signalling pathway, as well as high *ERBB2* mRNA and HER2 protein levels that represent therapeutic targets. This could explain the high accuracy obtained from the HER2‐driven morphometric signature model in predicting the response of HER2‐positive BC patients to HER2 pathway targeting therapy, superseding the HER2 IHC score‐based risk of recurrence model. The value of the combined HER2‐E sub‐type with *ERBB2* mRNA expression levels, and better prediction response to anti‐HER2 therapy was previously reported [8, 75].

The accuracy of the risk of recurrence model was improved through combining both ER and PR morphometric features. This agrees with several clinical trials and meta‐analyses that demonstrated a strong association between HR status and pCR [9, 59, 76] as well as patients outcomes [42].

HER2 intra‐tumour heterogeneity is a well‐known phenomenon in BC, defined as the coexistence of subpopulations of tumour cells with different HER2 clones. This has been reported in up to 40% of BC and more frequently associated with HR positivity, poor prognosis and was proposed to be a potential mechanism for anti‐HER2 resistance [77, 78]. This was obvious in the current study where HER2‐positive/ER‐positive tumours were significantly associated with high intra‐tumour variance compared to HER2‐positive/ER‐negative tumours.

Recent advances in digital pathology and AI have empowered us to analyse fine subcellular architectural and morphometric characteristics of tumours, offering a more comprehensive understanding of the disease. Previous studies that employed AI algorithms to predict HER2 status and response to therapy exhibited promising results [1, 21, 22, 23, 24, 25, 26, 27, 28]. However, the majority of these algorithms utilised an unsupervised data analysis approach to predict HER2 positivity rather than oncogenic signalling activity, and also lack clear explainability of the used morphological features and their clinical relevance. In contrast, our approach, which employed a biologically relevant and explainable set of morphometric features, could be used as a complementary tool for training AI algorithms in order to obtain more reproducible results.

Our study had some limitations, including the small number of external test cohorts, but we were limited by the cases with HER2‐E PAM50 molecular sub‐typing in the TCGA dataset. We believe that the data in this work could be validated on a larger cohort and even used for training of deep learning algorithms.

In conclusion, tumour morphometric features could reflect *HER2* oncogenic activity in BC and hence response to HER2 pathway targeting therapy. The cross‐talk between ER and HER2 impacted HER2 signalling activity and was reflected in the HER2‐driven morphometric features and intra‐tumour heterogeneity. HER2 IHC 3+/ER‐negative tumours were highly enriched in the HER2‐driven signature among HER2‐positive tumours. In clinical practice, the developed HER2‐morphometric signature is a robust tool that could be used as a prognostic and a predictive indicator of HER2 oncogenic signalling activity, aggressive tumour behaviour and hence the response to HER2 pathway targeting therapy.

## Author Contributions

**N. M. Atallah:** conceptualization (lead), data curation (lead), formal analysis (lead), investigation (lead), methodology (lead), writing – original draft (lead), writing – review and editing (lead). **S. Makhlouf:** validation (equal), visualization (equal). **M. Nabil:** software (equal), validation (equal). **A. Ibrahim:** validation (equal). **M. S. Toss:** writing – review and editing (equal). **N. P. Mongan:** supervision (equal). **E. Rakha:** supervision (equal), writing – review and editing (equal).

## Ethics Statement

This study was approved by the Yorkshire & the Humber—Leeds East Research Ethics Committee (REC Reference: 19/YH/0293) under the IRAS Project ID: 266925. Data collected were fully anonymised. A written informed consent was obtained from all patients in the study.

## Conflicts of Interest

The authors declare no conflicts of interest.

## Supporting information


Appendix S1

Appendix S2

## Data Availability

All data used in this study are available and can be accessed upon reasonable request. The following publicly available datasets were used on https://identifiers.org/cbioportal:brca_tcga.
