# Supplementary material for: Characterisation of HER2‐Driven Morphometric Signature in Breast Cancer and Prediction of Risk of Recurrence
Source: Cancer Med. 2025 Apr 17;14(8):e70852. doi: 10.1002/cam4.70852 (PMC12004275; doi:10.1002/cam4.70852)
Supplement: Supplementary file 1 — Appendix S1 [file CAM4-14-e70852-s002.docx]

**Supplementary File 1:**

1. **Morphometric digital image analysis**

The whole slide image (WSI) of 360 BC cases (both discovery and test cohorts) were analysed using the QuPath image analysis software version 4.3. which automatically perform a set of cellular and subcellular morphometric measurement. The following steps were considered during the image analysis process:

- *Image pre-processing:*

Colour normalization and stain separation using colour deconvolution were carried out as previously published. This was performed to obtain more accurate calculation of H&E stain optical density (OD) within tumour cells and for better cellular segmentation.

- *Cellular and nuclei segmentation and classification*

This was carried out using Cellpose and Stradist extensions within QuPath. For each case, 3 different areas (2000x2000 pixels each) were annotated. Areas selection was based on the highest tumour cellularity within the WSI which was identified through the density maps algorithm functionality within the QuPath. The model semantically segmented both cells and nuclei. An object classifier was trained using several annotated nuclei from different slides on differentiating tumour and immune cells and the trained classifier was then applied to all cases. The cellular and subcellular measurements identified by the model included nuclear dimensions (area, length, diameter), nuclear colour density, cell area, cytoplasmic area, nuclear/cytoplasmic ratio (N/C ratio) and nuclear/cell ratio. Features related to nuclear circularity were excluded as the Stardist tool adds smoothing features to the nuclear membrane which affect the accuracy of the measurements.

- *Spatial distribution and arrangement of tumour cell*

Delaunay 2D model was applied for spatial distribution and tumour cell arrangement assessment (1, 6). Delaunay triangulation is a geometric method used to partition a set of points (nuclei) in a plane (usually in 2D space) into non-overlapping triangles. The key characteristic of a Delaunay triangulation is that no nuclei in the dataset is inside the circumcircle (the circle that passes through all three vertices) of any triangle in the triangulation. Delaunay mean distance is a measure of average spacing or separation between nuclei in WSI. It can provide insights into the spatial characteristics of the tumour nuclei.

1. **HER2 morphometric signature and prognostic index**
2. XGBoost model is an ensemble machine learning algorithm that is widely used to handle tabular data and excels in predictive modelling, classification, and regression tasks. XGBoost works by training a number of decision trees. Each tree is trained on a subset of the data, and the predictions from each tree are combined to form the final prediction (52). We studied each feature contribution into the final prediction by applying Recursive Feature Elimination (RFE) which is an iterative algorithm that systematically removes less important features from the dataset based on model feature importance.


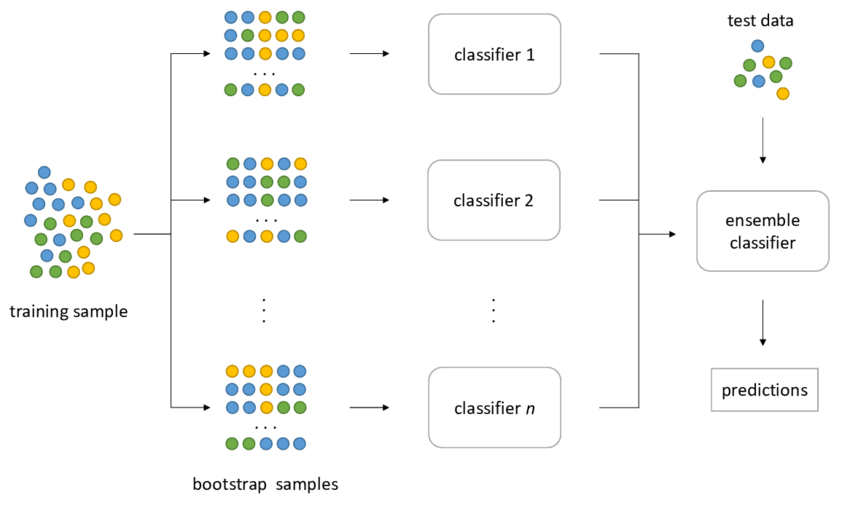


XGBoost architecture: It’s an ensemble of classifiers where each classifier is focused on the mistakes made with the previous classifier.

***Inputs***

- **Training dataset:**
- $D=\{(X_{i},y_{i}){\}}_{i=1}^{n}$with feature vectors $X_{i}$ (feature combination) and binary labels $y_{i}$ (HER2 or NON-HER2)
- **Test dataset:** $D_{\text{test}}=\{X_{j}{\}}_{j=1}^{m}$
- **Configuration for reproducibility:**

| Hyperparameter | Description | Value |
| --- | --- | --- |
| $\boldsymbol{\eta}$ | Step size shrinkage to prevent overfitting. Shrinks feature weights after each boosting step. | 0.3 |
| d | Maximum tree depth. Higher values increase complexity and overfitting risk. | 6 |
| $\boldsymbol{w}_{\boldsymbol{min}}$ | Minimum sum of instance weight (hessian) required in a child node. | 1 |
| subsample $\boldsymbol{s}$ | Fraction of training instances sampled for each boosting iteration. | 1 |
| scale_pos_weight | Balances positive and negative weights for imbalanced data. | 9 |

***Initialize Model Parameters***

Define the XGBoost binary cross entropy objective function:

$$\mathcal{L(}\theta)=\sum_{i=1}^{n} l(y_{i},\hat{y}_{i})$$

*Where, $\theta$ are the learnable weights.*

***Training***

1. Construct an ensemble of decision trees $\{f_{t}(X){\}}_{t=1}^{T}$
2. For each boosting iteration $t$

- Compute the first order and second-order gradients:

$$g_{i}^{(t)}=\frac{\partial l(y_{i},\hat{y}_{i}^{(t-1)})}{\partial\hat{y}_{i}}$$

$$h_{i}^{\left( t \right)}=\frac{\partial^{2}l\left( y_{i},\hat{y}_{i}^{\left( t-1 \right)} \right)}{\partial\hat{y}_{i}^{2}}$$

- Fit a new regression tree $f_{t}(X)$ to minimize the following objective:

$$\sum_{i=1}^{n} \left[ g_{i} f_{t} ( X_{i} ) + \frac{1}{2} h_{i} f_{t}^{2} ( X_{i} ) \right]$$

- Update predictions

$$\hat{y}_{i}^{(t)}=\hat{y}_{i}^{(t-1)}+\eta f_{t}(X_{i})$$

***Predictition***

1. Compute trained model output for test samples

$$\hat{y}_{j}=\sum_{t=1}^{T} \eta f_{t}\left( X_{j} \right) | j=1\ldots m$$

2. Apply the sigmoid activation function:

$$P(y_{j}=1\mid X_{j})=\frac{1}{1+e^{-\hat{y}_{j}}}$$

3. Convert probabilities to binary labels using threshold $\tau=0.5$


$$\hat{y}_{j}=\left\{ \begin{matrix} 1, & P(y_{j}=1\mid X_{j})\geq0.5 \\ 0, & \text{otherwise} \end{matrix} \right.$$

4. Survival analysis is a type of regression problem, but with a twist. It differs from traditional regression by the fact that parts of the training data can only be partially observed as they are censored.

Formally, each patient record consists of a set of covariates $x\in\mathbb{R}^{d}$, and the time $t>0$ when an event occurred or the time $c>0$ of censoring. Since censoring and experiencing and event are mutually exclusive, it is common to define an event indicator $\delta\in\{0;1\}$ and the observable survival time $y>0$. The observable time $y$ of a right censored sample is defined as

$$y=\min\left( t, c \right)=\left\{ \begin{aligned} t if \delta=1 \\ c if \delta=0 \end{aligned} \right.$$

For our model we used survival gradient boost (SGM) model with the partial likelihood loss of Cox’s proportional hazards model. Therefore, the objective is to maximize the log partial likelihood function, but replacing the traditional linear model $x^{T}\beta$ with the additive model $f(x)$:

$$arg\min_{f} \sum_{i=1}^{N} \delta_{i}\left[ f\left( x_{i} \right)-\log\left( \sum_{j\in R_{i}} e^{f\left( x_{j} \right)} \right) \right]$$

Rather than predicting a single point in time of an event, the prediction step in survival analysis focuses on predicting a function: either the survival or hazard function. The survival function S(t) returns the probability of survival beyond time, whereas the hazard function h(t) denotes an approximate probability that an event occurs in the small-time interval under the condition that an individual would remain event-free up to time.

For evaluation of the model accuracy, we used the concordance index (c-index). It is a measure of rank correlation between predicted risk scores $\hat{f}$ and observed time points $y.$ We also used time-dependent ROC curve. Given a time point $t$, we can estimate how well our model can distinguish subjects who will experience an event by time $t$ (sensitivity) from those who will not (specificity). The final AUC score is the mean score of the cumulative/dynamic area under the ROC at a given list of time points.
