# Supplementary material for: Characterisation of HER2‐Driven Morphometric Signature in Breast Cancer and Prediction of Risk of Recurrence
Source: Cancer Med. 2025 Apr 17;14(8):e70852. doi: 10.1002/cam4.70852 (PMC12004275; doi:10.1002/cam4.70852)
Supplement: Supplementary file 2 — Appendix S2 [file CAM4-14-e70852-s001.docx]

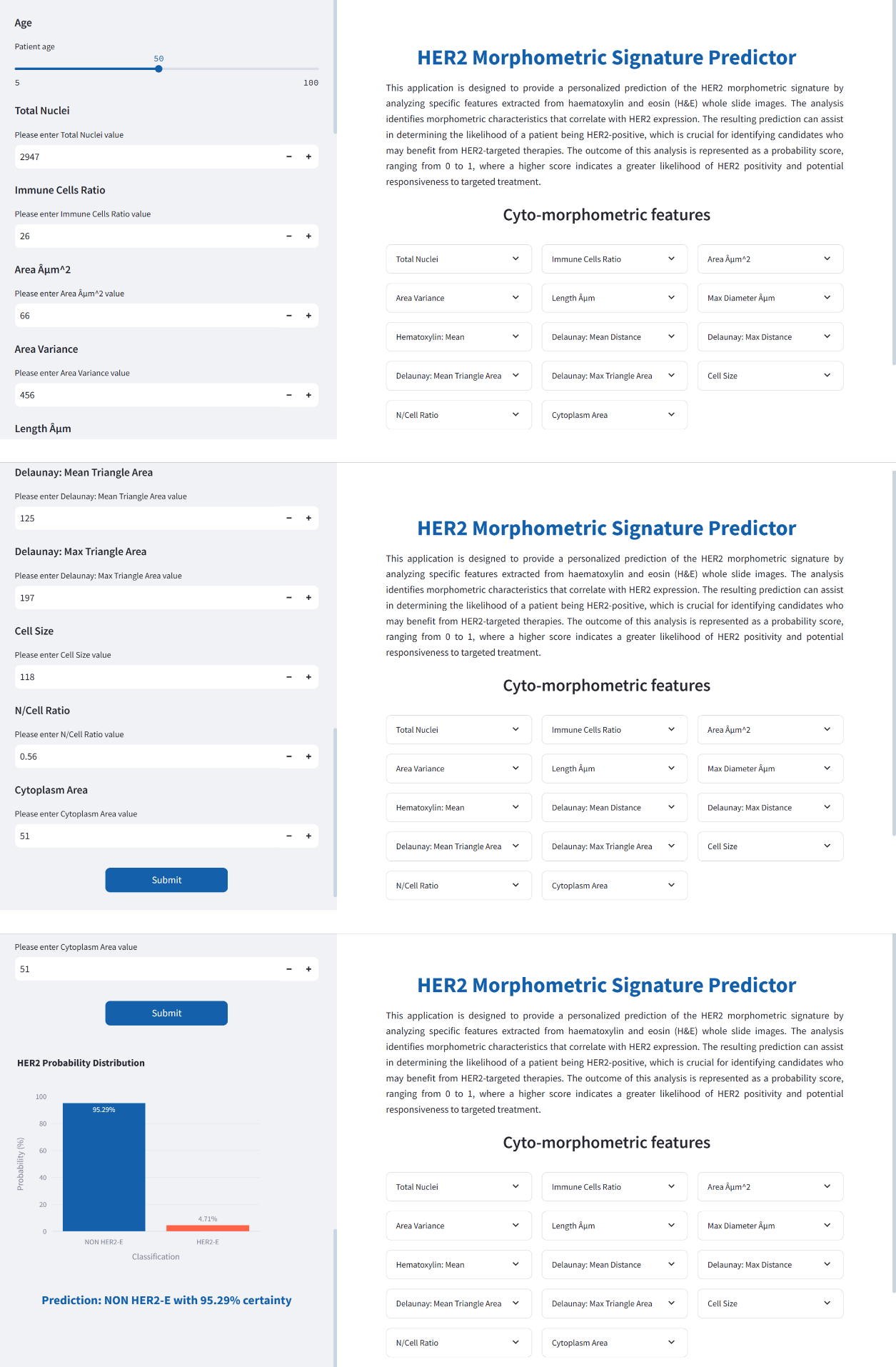


Supplementary Figure 1: Screenshots from HER2-morphometric signature web tool


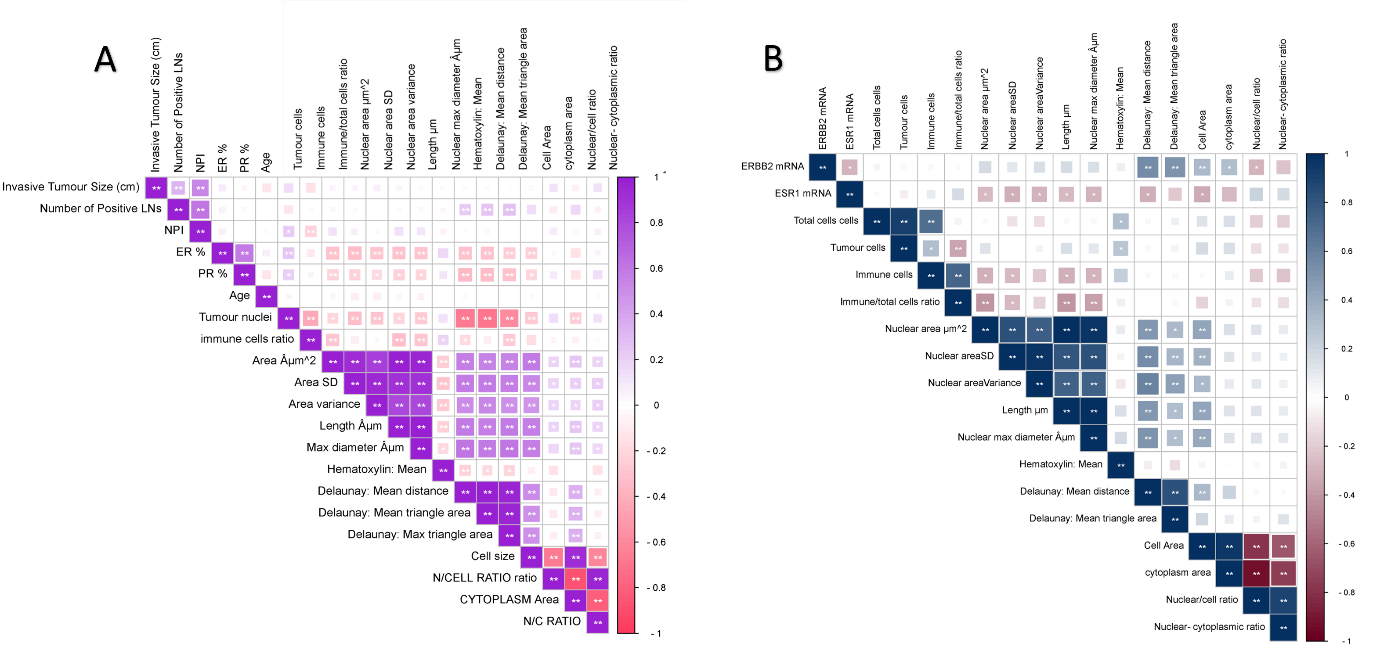


Supplementary Figure 2: A correlation matrix demonstration the correlation between the significant HER2 morphometric features and tumour clinicopathological parameters (A) and both *ERBB2* mRNA and *ESR1* mRNA expression level in HER2-positive tumours (B). * Represents significant p value<0.05, ** when p value <0.01.


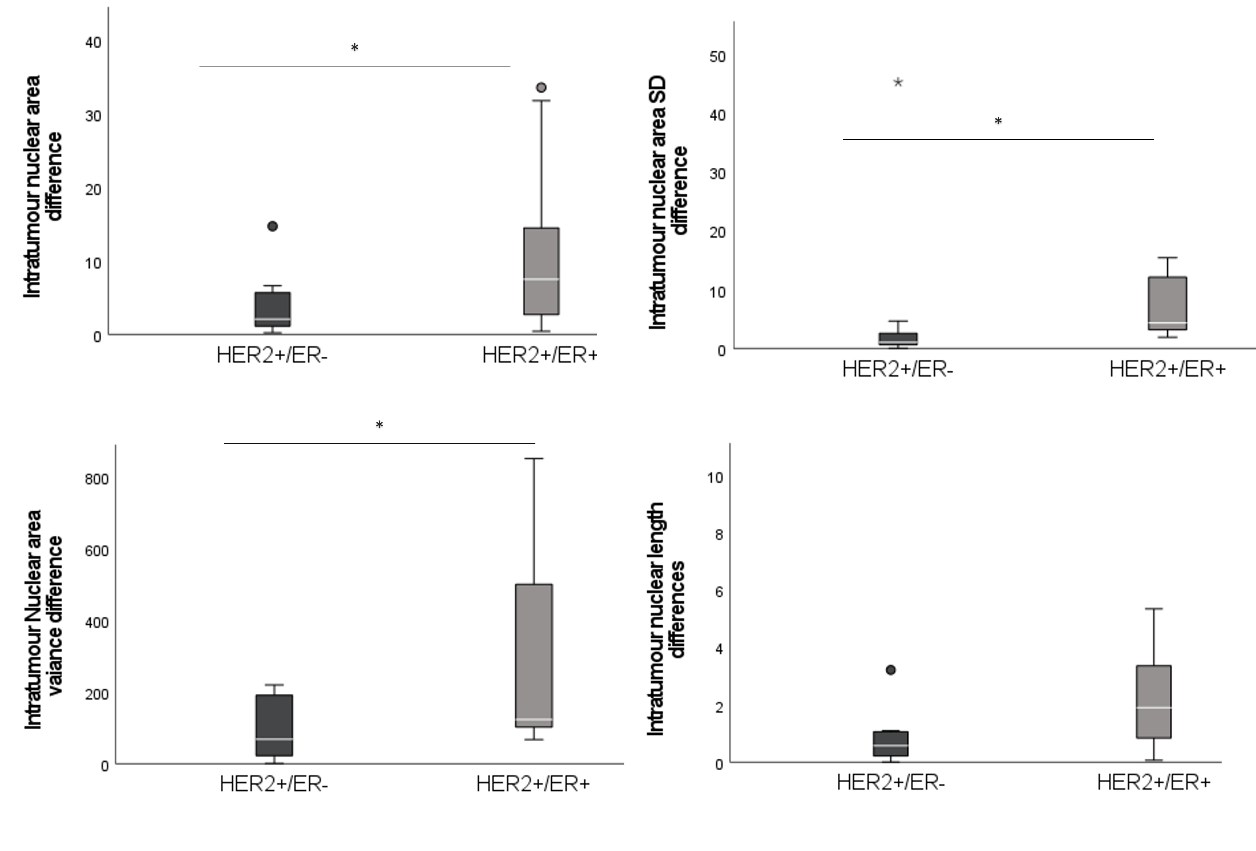
Supplementary Figure 3 : Box blot chart demonstrating intratumor heterogeneity represented by feature variance among annotated areas in HER2+ cases adjusted for ER status. HER2+: HER2-positive; ER-positive: Oestrogen receptor-positive; ER-: ER-negative


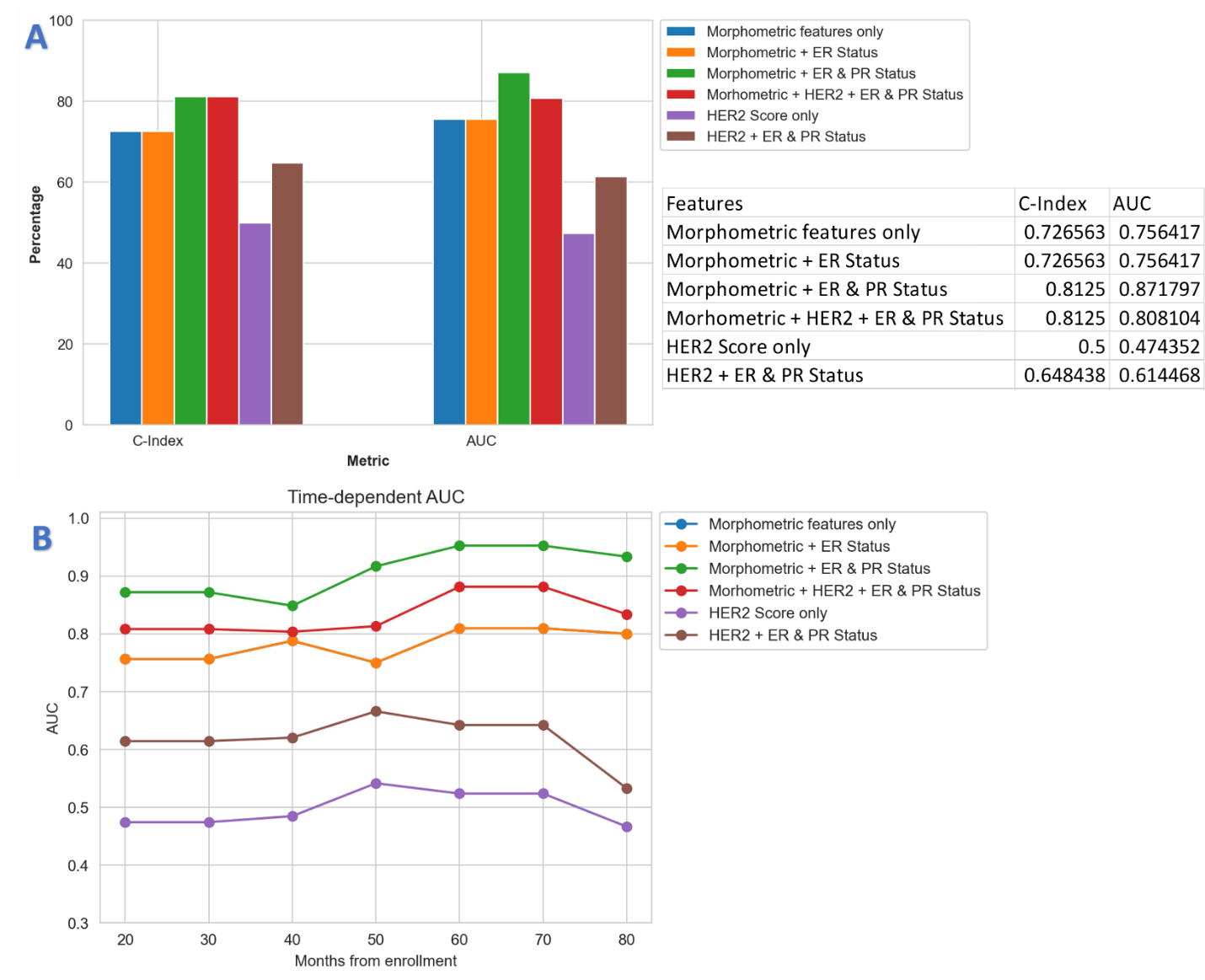


Supplementary Figure 4: HER2 risk of recurrence prognostic index. A: A bar chart demonstrating the average accuracy of each prognostic index in the test cohort. B: A time dependent ROC curve highlighting the AUC of each index at a given time point. Both charts show that “morphometric features +ER &PR status “is the most accurate index.

Supplementary Table 1: Summary of the extracted morphometric features using digital image analysis software

|  | Cyto-morphometric Feature | Description | Mean | | | | |  |  |
| --- | --- | --- | --- | --- | --- | --- | --- | --- | --- |
|  |  |  | HER2-negative | | HER2-positive | | |  |  |
| Quantitative criteria | Average total detected nuclei/case | Total number of segmented nuclei in 3 annotated areas | 38834 | | 30730 | | |  |  |
|  | Tumour nuclei/case | Average number of tumour nuclei detected within annotated areas by the object classifier algorithm | 2577 | | 2080 | | |  |  |
|  | Immune cells/case | Average number of immune cell nuclei detected within annotated areas by the object classifier algorithm | 1278 | | 987 | | |  |  |
|  | Immune cells/tumour cells | The ratio of immune cell nuclei to the total detected nuclei | 31.0 | | 16.6 | | |  |  |
| Nuclear Dimensions | Nuclear area µm 2 | The area occupied by single segmented tumour cell nuclei using Stardist algorithm | 55.2 | | 60.8 | | |  |  |
|  | Nuclear area standard deviation µm* | The dispersion of segmented nuclei area in relation to the mean. Low, or small, standard deviation indicates data are clustered tightly around the mean, and high, or large, standard deviation indicates data are more spread out. | 19.1 | | 22.0 | | |  |  |
|  | Nuclear area variance* | Variance is the average squared deviations of nuclear areas from the mean | 406.9 | | 570.1 | | |  |  |
|  | Nuclear length (perimeter) µm | The length of single cell nuclear membrane | 27.1 | | 28.4 | | |  |  |
|  | Nuclear max diameter µm | Maximum diameter of single cell nuclear membrane | 10.0 | | 10.4 | | |  |  |
|  | Nuclear min diameter µm | Minimum diameter of single cell nuclear membrane | 7.0 | | 7.3 | | |  |  |
| Nuclear basophilia | Nuclear Haematoxylin: Mean | Mean nuclear Haematoxylin optical density in single tumour cell | 0.49 | | 0.38 | | |  |  |
|  | Nuclear Haematoxylin: Median | Median nuclear Haematoxylin optical density in single tumour cell | 16.1 | | 17.9 | | |  |  |
|  | Nuclear Haematoxylin: Min | Minimum nuclear Haematoxylin optical density in single tumour cell | 10.6 | | 11.5 | | |  |  |
|  | Nuclear Haematoxylin: Max | Maximum nuclear Haematoxylin optical density in single tumour cell | 23.8 | | 27.0 | | |  |  |
|  | Nuclear Haematoxylin: Standard deviation | Nuclear Haematoxylin optical density standard deviation in single tumour cell | 0.24 | | 0.20 | | |  |  |
| Spatial distribution analysis | Delaunay: Mean distance | It is the mean of distance where no point (nuclei) lies within the circumcircle of any line formed. Larger distance means that the tumour nuclei are widely separated from each other (less overlap) | | 16.6 | | | 18.6 | |  |
|  | Delaunay: Median distance | It is the median of distance where no point (nuclei) lies within the circumcircle of any line formed | | 16.1 | | | 18.0 | |  |
|  | Delaunay: Max distance | It is the maximum of distance where no point (nuclei) lies within the circumcircle of any line formed | | 23.8 | | | 27 | |  |
|  | Delaunay: Min distance | It is the minimum of distance where no point (nuclei) lies within the circumcircle of any line formed | | 10.6 | | | 11.6 | |  |
|  | Delaunay: Mean triangle area | It is the mean of triangulated network area where no point (nuclei) lies within the circumcircle of any triangle formed. Larger area means that the tumour nuclei are widely separated from each other (less overlap) | | | | 108.1 | | 134.0 | |
|  | Delaunay: Max triangle area | It is the maximum of triangulated network area where no point (nuclei) lies within the circumcircle of any triangle formed | | | | 171.1 | | 213.5 | |
| Tumour cluster Nuclear Dimensions | Cluster mean: Nuclear area µm^2^ | The area occupied by the segmented tumour cell nuclei cluster | | | | 54.7 | | 57.9 | |
|  | Cluster mean: Nuclear length µm | The length of nuclear membrane measured in tumour cluster | | | | 26.9 | | 27.4 | |
|  | Cluster mean: Nuclear max diameter µm | Maximum diameter of nuclear membrane measured in tumour cluster | | | | 9.9 | | 10.1 | |
|  | Cluster mean: Nuclear min diameter µm | Minimum diameter of nuclear membrane measured in tumour cluster | | | | 7.0 | | 7.1 | |
| Tumour cluster Nuclear basophilia | Cluster mean: Nuclear Haematoxylin: Mean | Mean nuclear Haematoxylin optical density measured in tumour cluster | | | | 0.49 | | 0.39 | |
|  | Cluster mean: Nuclear Haematoxylin: Median | Median nuclear Haematoxylin optical density measured in tumour cluster | | | | 0.42 | | 0.37 | |
|  | Cluster mean: Nuclear Haematoxylin: Min | Minimum nuclear Haematoxylin optical density measured in tumour cluster | | | | 0.016 | | 0.006 | |
|  | Cluster mean: Nuclear Haematoxylin: Max | Maximum nuclear Haematoxylin optical density measured in tumour cluster | | | | 1.18 | | 1.1 | |
|  | Cluster mean: Nuclear Haematoxylin: Std.Dev. | Nuclear Haematoxylin optical density standard deviation measured in tumour cluster | | | | 0.24 | | 0.17 | |
| Tumour cluster Spatial distribution analysis | Cluster mean: Delaunay: Mean distance | It is the mean of distance where no point (nuclei) lies within the circumcircle of any line formed, calculated for tumour cluster | | | | 16.6 | | 18.5 | |
|  | Cluster mean: Delaunay: Median distance | It is the median of distance where no point (nuclei) lies within the circumcircle of any line formed, calculated for tumour cluster | | | | 16.12 | | 17.8 | |
|  | Cluster mean: Delaunay: Max distance | It is the maximum of distance where no point (nuclei) lies within the circumcircle of any line formed, calculated for tumour cluster | | | | 23.8 | | 27.5 | |
|  | Cluster mean: Delaunay: Min distance | It is the minimum of distance where no point (nuclei) lies within the circumcircle of any line formed, calculated for tumour cluster | | | | 10.6 | | 11.2 | |
|  | Cluster mean: Delaunay: Mean triangle area | It is the mean of triangulated network area where no point (nuclei) lies within the circumcircle of any triangle formed for the whole tumour cluster. Larger area means that the tumour nuclei are widely separated from each other (less overlap) | | | | 107.9 | | 132.2 | |
|  | Cluster mean: Delaunay: Max triangle area | It is the maximum of triangulated network area where no point (nuclei) lies within the circumcircle of any triangle formed, calculated in tumour clusters | | | | 170.4 | | 219.9 | |
|  | Cell Area µm^2^ | The mean area of cells detected using Cellpose algorithm | | | | 100.6 | | 123.0 | |
|  | Nuclear/Cell ratio | The ratio of nuclear area to cell area | | | | 0.54 | | 0.51 | |
|  | Cytoplasm area µm^2^ | cell area-nucleus area | | | | 49.8 | | 62.1 | |
|  | Nuclear/Cytoplasmic ratio | The ratio of nuclear to cytoplasmic areas | | | | 1.2 | | 1.1 | |

Supplementary Table 2: Pathway enrichment analysis of HER2-morphometric signature positive vs HER2-morphometric signature negative cases

| Gene Set | Description | Size | FDR |
| --- | --- | --- | --- |
| hsa01522 | Endocrine resistance | 98 | 0.0028299 |
| hsa05224 | Breast cancer | 147 | 0.0048011 |
| hsa05205 | Proteoglycans in cancer | 201 | 0.0081827 |
| hsa05213 | Endometrial cancer | 58 | 0.027888 |
| hsa05223 | Non-small cell lung cancer | 66 | 0.027888 |
| hsa04917 | Prolactin signalling pathway | 70 | 0.027888 |
| hsa01521 | EGFR tyrosine kinase inhibitor resistance | 79 | 0.030446 |
| hsa04012 | ErbB signalling pathway | 85 | 0.030836 |
| hsa05215 | Prostate cancer | 97 | 0.035670 |
| hsa05200 | Pathways in cancer | 526 | 0.042924 |
